# Habitat suitability and active component responses of *Eleutherococcus senticosus* under climate change: implications for conservation and wild tending

**DOI:** 10.3389/fpls.2026.1871650

**Published:** 2026-09-14

**Authors:** Detai Duan, Hengjun Xu, Mengsha You, Mengjiao Li, Jianxi Chen, Boyan Zhang, Bingrui Chen, Xiaoxia Jin, Xinxin Zhang

**Affiliations:** 1 Heilongjiang Research Center of Genuine Wild Medicinal Materials Germplasm Resources, School of Life Sciences and Technology, Harbin Normal University, Harbin, China; 2 The College of Forestry, Beijing Forestry University, Beijing, China

**Keywords:** conservation management, eleutherococcus senticosus, fingerprint chromatogram, global change, targeted wild tending areas

## Abstract

**Introduction:**

*Eleutherococcus senticosus* (Rupr. & Maxim.) Maxim. is an important medicinal shrub with increasing demand for its wild resources. However, the responses of its future habitat suitability and medicinal quality to global environmental change remain poorly understood.

**Methods:**

Future climate projections under multiple Shared Socioeconomic Pathway (SSP) scenarios, land-use dynamics, HPLC fingerprint data, and ensemble species distribution models were integrated to evaluate the habitat suitability, medicinal quality, and conservation priorities of *E. senticosus* in Heilongjiang Province.

**Results:**

Climate variables, particularly Bio18, Bio11, and Bio08, were identified as the dominant drivers of habitat suitability, whereas land-use change exerted comparatively limited effects on habitat dynamics. Future climate change was projected to expand the overall suitable habitat of E. senticosus in Heilongjiang Province while reducing the extent of highly suitable habitats. The areas with high contents of the five major bioactive compounds, namely coniferin, syringin, eleutheroside E, isofraxidin, and syringaresinol-4′-O-β-D-glucopyranoside, were all projected to increase under future climate scenarios. Priority conservation analysis based on habitat suitability showed that existing nature reserves covered only 12.899% of the identified priority conservation areas. Wild tending priority areas, identified by integrating habitat suitability and medicinal component distributions, were mainly concentrated in the Lesser Khingan Mountains under current climate conditions but were projected to shift toward the western Lesser Khingan Mountains under future climate change.

**Discussion:**

These findings provide theoretical support for the conservation, wild tending, standardized management, and sustainable utilization of *E. senticosus*.

## 1 Introduction

Plants are a core component of global biodiversity, providing fundamental goods and services for human society (Haswell, 2024; Pironon et al., 2024). Understanding their geographic distribution is therefore essential for the sustainable management of plant resources (Pironon et al., 2024). However, increasing resource consumption and other anthropogenic pressures have placed growing threats on plant diversity (Díaz et al., 2019). Among these, medicinal plants are particularly vulnerable because of their high market demand and economic value, facing habitat loss and overharvesting (Masood, 1997; Kling, 2016). Approximately 70%-90% of medicinal and aromatic plants are still harvested from the wild (Kling, 2016), and nearly 15,000 medicinal plant species are currently threatened by overexploitation and habitat destruction (Chen et al., 2016). To alleviate the conflict between resource conservation and medicinal demand, cultivation approaches such as wild tending and ecological planting have been increasingly promoted (Miao et al., 2024). However, these practices often face a trade-off between yield and medicinal quality, as the accumulation of bioactive compounds is strongly influenced by environmental conditions (Guo et al., 2021). The distinct geographic distribution patterns of wild medicinal plants reflect their long-term adaptation to local biotic and abiotic environments (Davis and Choisy, 2024). Therefore, identifying suitable habitats and their environmental drivers is fundamental for conservation, cultivation planning, and sustainable utilization.

The distribution dynamics of plant species have been continuously shaped by climate change throughout Earth’s evolutionary history (Levin, 2019; Trew and Maclean, 2021). However, anthropogenic climate change is occurring at an unprecedented rate (Sala et al., 2000; Bush et al., 2004; Trew and Maclean, 2021). Numerous studies have demonstrated that climate change is rapidly altering plant distribution patterns worldwide (Dyderski et al., 2018; Chen et al., 2023; Franklin, 2023; Xu et al., 2023). For example, *Quercus mongolica*, a representative temperate tree species in eastern Asia, has shifted northward in response to recent climate warming (Tang et al., 2024). Compared with dominant tree species that possess relatively well-defined climatic niches, however, the responses of medicinal plants to future climate change remain poorly understood. Besides climate change, land-use and land-cover change (LUCC) is another major driver of biodiversity loss that can directly reduce suitable habitats and indirectly limit species adaptation by increasing habitat fragmentation and restricting gene flow (Jump and Peñuelas, 2005; Fischer and Lindenmayer, 2007). Because climate change and LUCC often interact to reshape species distributions (Sala et al., 2000; Chen et al., 2023), integrating both drivers is essential for accurately predicting the future distribution of medicinal plants. Although species distribution models have been widely applied to assess the effects of climate change on plant distributions, most previous studies on medicinal plants have focused primarily on habitat suitability. Far fewer studies have simultaneously considered future habitat dynamics, medicinal quality, and conservation planning under global change.

*Eleutherococcus senticosus* (Rupr. & Maxim.) Maxim. (Araliaceae) is a deciduous shrub primarily distributed in northeastern Asia, with the temperate forests of China representing its core range (Editorial Committee of Flora of China, Chinese Academy of Sciences, 1993). Its dried roots, rhizomes, and stems have long been used in traditional Chinese medicine (Committee National Pharmacopoeia, 2020). Modern pharmacological studies have demonstrated multiple therapeutic activities, including improving sleep, learning and memory, alleviating anxiety and depression, enhancing microcirculation, regulating blood glucose, and exhibiting antitumor effects. Owing to weak sexual reproduction and limited vegetative propagation in wild populations, *E. senticosus* is listed in the China Plant Red Data Book: Rare and Endangered Plants and is protected as a national Class III wild medicinal plant in China (Wei et al., 2024). Increasing market demand, coupled with continued reliance on wild harvesting, has caused severe depletion of natural populations, while artificial cultivation remains constrained by low survival and productivity, resulting in a widening gap between supply and demand (Guo et al., 2021; Liu et al., 2023). Previous ecological suitability assessments of *E. senticosus* have several limitations, including species distribution modeling was not performed across the entire ecological niche of the species, an overemphasis on climatic variables while overlooking soil and biotic factors, reliance on single climate models for future projections, and a lack of uncertainty assessment (Wan et al., 2016; Xue et al., 2026). Furthermore, under global change, the combined effects of climate, soil, and habitat conditions on both species distribution and medicinal quality remain poorly understood.

Taking *E. senticosus* populations in Heilongjiang Province as the research object, this study conducted systematic field sampling, collected samples of medicinal materials of *E. senticosus* from different regions, and obtained species distribution records at the global scale. Methodologically, this study integrated high-performance liquid chromatography fingerprint data, future climate projection and land use dynamic data under multiple Shared Socioeconomic Pathways, constructed an ensemble modeling framework of multiple species distribution models based on the biomod2 platform, and simultaneously explored the quantitative relationship between environmental variables and active ingredient content via all-subsets regression analysis. Accordingly, this study pursued three specific objectives: (1) to evaluate how the distribution of suitable habitats and medicinal quality of *E. senticosus* may change under future climate and land-use scenarios; (2) to identify the key environmental drivers underlying habitat suitability and bioactive compound accumulation in *E. senticosus*; (3) to identify priority areas for wild resource conservation and wild tending of *E. senticosus* under future climate and land-use scenarios.

## 2 Materials and methods

### 2.1 Collection of samples of *E. senticosus* plants and acquisition of distribution data

To ensure that the chemical composition profiles of *E. senticosus* comprehensively characterize the plant’s properties representative of Heilongjiang Province, we conducted a systematic large-scale investigation across its natural distribution areas (within forest habitats) in Heilongjiang Province. The medicinal materials of *E. senticosus* were collected and dried, and a total of 36 samples were obtained for subsequent component determination (**Figures 1A, B**).

**Figure 1:**
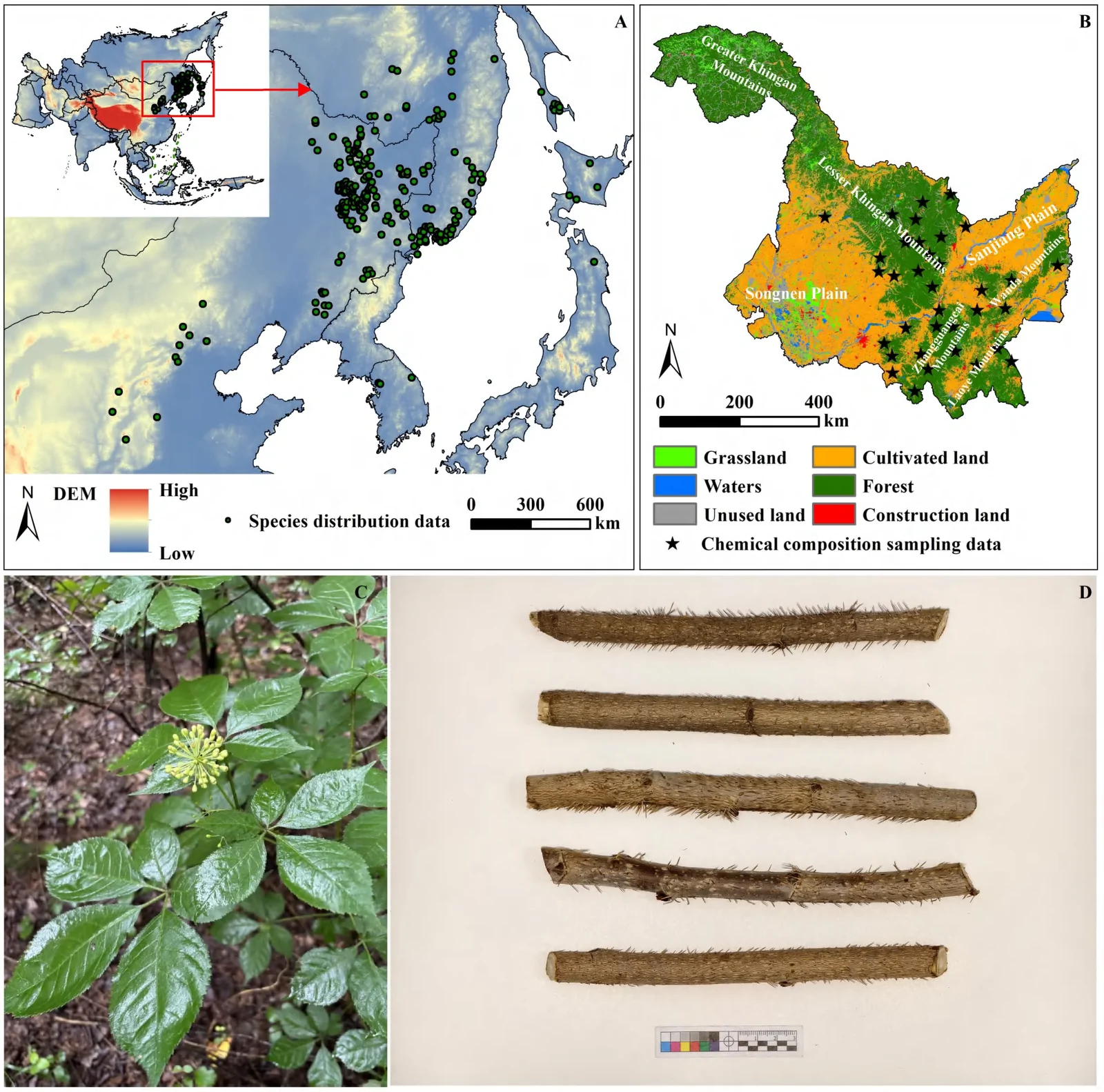
Geographical distribution and sampling locations of *E. senticosus.* **(A)** Geographical distribution points of *E. senticosus*; **(B)** Sampling sites for chemical composition analysis of *E. senticosus*; **(C)** Plant specimens of *E. senticosus*; **(D)** Medicinal materials of *E. senticosus*.

This study obtained the geographical distribution data of *E. senticosus* through three approaches: (1) Field Survey: Comprehensive on-site investigations were conducted from 2017 to 2025, during which GPS equipment was utilized to precisely record the geographical coordinates of *E. senticosus*. (2) Database Retrieval: Utilizing the plantR package in R (De Lima et al., 2023) and online database resources, geo-distribution data of *E. senticosus* were systematically collected for the period from 2000 to 2025, and an accuracy validation process was implemented for the associated photographic records. (3) Literature Review: Relevant publications were retrieved from academic databases including Google Scholar and the China National Knowledge Infrastructure (CNKI), from which geographical coordinates of *E. senticosus* documented therein were extracted. To eliminate abnormally distributed data, the original coordinates were first screened using Google Earth Pro software (Google Earth USA) to remove points located in non-suitable habitats for *E. senticosus* such as construction land, water bodies, farmland, and grassland. Subsequently, the spThin package in R was used for spatial redundancy removal, deleting clustered points within a 10 km range. To ensure consistency with the objective of assessing wild populations, occurrence records originating from artificial cultivation sites were excluded, and only records representing natural populations were retained for subsequent analyses. Through the aforementioned two-step data cleaning process, the spatial autocorrelation of *E. senticosus* geographic distribution data was significantly mitigated (Aiello-Lammens et al., 2015), resulting in the retention of 229 valid geographical distribution points for subsequent modeling analysis (**Figure 1A**).

### 2.2 Determination of effective chemical components of *E. senticosus*

To ensure the representativeness of the experimental results, this study employed 15 distinct batches of *E. senticosus* herbs for the construction of comparative chromatographic profiles. To ensure the experimental results have a certain degree of representativeness, a total of 15 batches of *E. senticosus* herbs were selected for the preparation of test solutions and chromatographic conditions for machine determination. The “Similarity Evaluation System for Chromatographic Fingerprints of Traditional Chinese Medicine (2012 Edition)” software was used, with a time window of 0.5 min; the method of generating the reference chromatogram was the mean method. Full-spectrum peak matching was conducted. After fitting, a total of 39 common characteristic peaks were obtained. Based on the chromatographic profiles of various samples, seven component peaks had been previously identified using reference standards, including protocatechuic acid, coniferin, syringin, eleutheroside B1, eleutheroside E, isofraxidin, and syringaresinol-4-O-*β*-D-glucopyranoside. In addition to these, four additional chromatographic peaks were selected as characteristic peaks, which exhibited good resolution, large peak area, and stable retention times.

The specific relative retention times of each characteristic peak relative to the reference peak of eleutheroside E (S-peak) for all 36 test samples are detailed in the Supporting Information Text. In the characteristic chromatograms of all 36 batches of test samples, 11 characteristic peaks were consistently detected, corresponding to the 11 characteristic peaks observed in the reference chromatogram of the reference material. Among these, Peak 2, Peak 3, Peak 4, Peak 6, Peak 7, Peak 8, and Peak 9 exhibited retention times identical to those of the reference standards for protocatechuic acid, coniferin, syringin, eleutheroside B1, eleutheroside E, isofraxidin, and syringaresinol-4’-O-*β*-D-glucopyranoside, respectively. Using Peak S corresponding to the reference material of eleutheroside E as the reference peak, the relative retention times of all characteristic peaks relative to Peak S deviated by no more than10% from the specified values. Thus, the characteristic chromatogram of the test sample meets the criteria. The selected characteristic peaks included major constituents documented in the Chinese Pharmacopoeia as well as compounds with well-established pharmacological activities (Committee National Pharmacopoeia, 2020). Moreover, chromatographic fingerprints can comprehensively reflect the consistency and stability of the overall chemical composition of medicinal materials. Therefore, these characteristic peaks provide a chemical basis for evaluating the medicinal quality and quality consistency of *E. senticosus*.

### 2.3 Sources of geospatial data

This study selected 26 environmental factors as analytical variables, covering four major categories: climate, soil, normalized difference vegetation index and human footprint (**Table 1**). To ensure that the environmental data can objectively reflect current climatic characteristics, we obtained monthly average minimum temperature, monthly average maximum temperature, and monthly average precipitation data from 2000 to 2018 from the World Climate Database (www.worldclim.org). Based on these data, we calculated 19 climate factors using the biovar package in R, with a time span also covering 2000 to 2018. Soil variables were sourced from the harmonized world soil database (HWSD V2.0, https://www.fao.org/). Following previous studies (Lin et al. (2024), five soil variables (Bulk density, Clay content, pH, Silt content, and soil organic carbon) were incorporated into the species distribution model of *E. senticosus*. Considering the growth characteristics and ecological requirements of *E. senticosus*, particularly its dependence on soil moisture availability and nutrient conditions within the root-accessible soil layer (Zhu et al., 1988), soil variables from the 0–40 cm depth were selected for model analysis. As HWSD v2.0 provides soil properties for two depth intervals (0–20 cm and 20–40 cm), the average values of these two layers were calculated to represent the integrated soil conditions within the main rooting zone of *E. senticosus*. Human footprint data were sourced from Mu et al (Mu et al., 2022). The NDVI data, which represents the average value for the period from July to September between 2000 and 2020, was downloaded from the MODIS (https://www.earthdata.nasa.gov). The original spatial resolutions were 2.5 arc-minutes for climate data and approximately 30 arc-seconds for soil, human footprint, and NDVI data. To ensure spatial consistency among environmental predictors, all environmental variables were resampled to a common spatial resolution of 2.5 arc-minutes prior to model construction.

**Table 1:** Predictive variables for construction of species distribution models.

Abbreviation	Full name	Units
Bio01	Annual mean temperature	°C
Bio02	Annual mean diurnal range	°C
Bio03	Isothermality	%
Bio04	Temperature seasonality	–
Bio05	Max temperature of warmest month	°C
Bio06	Min temperature of coldest month	°C
Bio07	Annual temperature range	°C
Bio08	Mean temperature of wettest quarter	°C
Bio09	Mean temperature of driest quarter	°C
Bio10	Mean temperature of warmest quarter	°C
Bio11	Mean temperature of coldest quarter	°C
Bio12	Annual precipitation	Mm
Bio13	Precipitation of wettest month	Mm
Bio14	Precipitation of driest month	Mm
Bio15	Precipitation seasonality (CV)	–
Bio16	Precipitation of wettest quarter	Mm
Bio17	Precipitation of driest quarter	Mm
Bio18	Precipitation of warmest quarter	Mm
Bio19	Precipitation of coldest quarter	Mm
Bulk	Topsoil reference bulk density	kg/dm^3^
Clay	Topsoil Clay Fraction	%
Ph	pH Value (H_2_O)	–
Silt	Topsoil Silt Fraction	%
Soc	Soil Organic Matter	%
Human	Human Footprint dataset from2000 to 2018	–
NDVI	Normalized difference vegetation index	–

To mitigate model overfitting induced by multicollinearity among environmental factors, this study employed a multi-step screening strategy to optimize the variable set. The initial distribution model was developed using the MaxEnt algorithm with 10 replicate runs (**Supplementary Table 1**), whereby the relative contribution rates of environmental variables to the distribution of *E. senticosus* were quantified and used as a preliminary criterion for variable optimization. Subsequently, the correlation analysis function of the ENMTools software was utilized to assess the correlations among the screened factors. For any pair of factors with an absolute value of the correlation coefficient |r| ≥ 0.8, the factor exhibiting a higher contribution rate, as determined by the MaxEnt model output, was retained. Finally, a variance inflation factor (VIF) test was performed, and variables with VIF values exceeding 5 were eliminated (Hellegers et al., 2020). A total of 13 environmental factors were ultimately selected for subsequent modeling and analysis.

In future environmental factor selection, to enhance the stability and reliability of model prediction outcomes, three widely utilized global circulation models (GCMs), namely CNRM-CM6-1, MIROC-ES2L, and MRI-ESM2-0, were selected. Each model encompasses three future periods (2050s, 2070s, 2090s) and four Shared Socioeconomic Pathways (SSPs) scenarios: SSP126 (low radiative forcing scenario), SSP245 (intermediate radiative forcing scenario), SSP370 (moderate-to-high radiative forcing scenario), and SSP585 (high radiative forcing scenario). Utilizing ArcMap 10.5 software, climate data from multiple models under the same temporal period and emission scenario were averaged to generate a standardized future climate dataset. Soil, NDVI and human footprint variables were assumed to remain constant and their current datasets were used in all future projections (Stanton et al., 2012).

To comprehensively evaluate the potential impacts of land use on the suitable habitats of *E. senticosus* in the context of climate change, this study set two hypotheses considering the uncertainty in the migration capacity of plant populations, which respectively represent the extreme scenarios of species’ responses to climate change: Supposition 1: It holds that during future climate change, the actual migration rate of *E. senticosus* populations can hardly keep pace with the spatial shift of climatically suitable areas. Therefore, new populations can only be established in the overlapping regions between the current climatically suitable areas and the climatically suitable areas of a specific future period. The core basis of this supposition is that the seed dispersal capacity and colonization rate of most plants are limited, and, coupled with the intensification of habitat fragmentation, actual migration often lags behind the movement of climate zones (Zani et al., 2024). Accordingly, the area of suitable habitat is defined as the forest coverage area within the intersection of climatically suitable areas in the current period and the target future period. The specific operation is as follows: overlay the raster of current climatically suitable areas and the raster of future climatically suitable areas, extract the overlapping pixels, then mask and clip using forest type layers in the corresponding land use data, and finally count the area of remaining pixels. Supposition: This supposition assumes that *E. senticosus* populations can respond rapidly to climate change, and fully migrate to new climatically suitable areas after future climate conditions change, relying on effective long-distance dispersal mechanisms. Based on an idealized scenario with no migration constraints, this supposition is commonly used to assess the potential maximum distribution range of species (Zani et al., 2023). Accordingly, the area of suitable habitat is defined as the total forest coverage area within the climatically suitable areas of the target future period. The specific operation is as follows: directly use the forest type raster in future land use data as a mask to clip the distribution raster of future climatically suitable areas, and then count the area of the obtained pixels. The habitat areas under the two hypotheses are all constrained by the same set of future land use scenarios.

The current (2020) land use data is derived from the MODIS dataset provided by the NASA Earth Data Platform (https://search.earthdata.nasa.gov/search/); the future land use projection data is obtained from the research results of Hou et al. (2022), which covers four scenarios consistent with climate change pathways: SSP126, SSP245, SSP370 and SSP585. The original spatial resolution of both current and future land use datasets is approximately 30 arc-seconds. By comparing the area change trends under the two hypotheses, the impact range of differences in migration capacity on habitat dynamics can be quantified, so as to provide a reference for risk boundaries in the formulation of conservation strategies. All land use data were resampled to a spatial resolution of 2.5 arc-minutes using ArcMap 10.5 software.

### 2.4 Statistical modeling and data analysis

#### 2.4.1 Construction of species distribution models

To elucidate the dynamics of *E. senticosus* population distribution patterns and the associated environmental processes under climate change, a species distribution model was employed to generate a probability distribution layer reflecting the species’ complete ecological niche. Specifically, utilizing the R package biomod2, ten distinct models were developed based on 229 species distribution data and environmental data under 13 distinct climate scenarios. All models other than the MaxEnt model utilized default parameters for simulating the distribution of *E. senticosus*. Before formal modeling, this study allocated 75% of the species distribution data as training data and the remaining 25% as test data (with 5 replicates). Modeling analyses were then conducted based on the number of pseudo-absence points, which were set to four common levels: equal to the number of occurrence records, 1000, 5000, and 10000. The results demonstrated that when the number of pseudo-absence points was set to 5000, The accuracy of most models and the mean precision of all models reach the maximum (**Supplementary Table 2**). Consequently, in this study, the pseudo-absence points were fixed at 5000 and the modeling process was repeated three times, yielding a total of 150 model results (10 models × 5 replicates × 3 groups of pseudo-existence points).

To optimize the MaxEnt model, the present study employed the kuenm package in R to evaluate model performance under various combinations of regularization multipliers (RM) and feature classes (FC) (Cobos et al., 2019). To optimize the MaxEnt model, this study combined 17 regularization multiplier values (0.1–1 at 0.1 intervals; 2–6 at 1 intervals; and 8, 10) with five feature classes: linear (L), quadratic (Q), hinge (H), product (P), and threshold (T). Based on these combinations, we constructed 527 candidate models. The optimal model was selected based on the delta values derived from the Akaike’s Information criterion corrected (AICc). The model with the minimum AICc value (ΔAICc = 0) is considered the optimal one (Cobos et al., 2019). In this study, the optimized parameters for the MaxEnt software were set as RM = 5 and FC = LQPTH.

The most precise approach for validating the predictive accuracy of Species Distribution Models (SDMs) involves a combined assessment that uses the True Skill Statistic (TSS) and the area under the receiver operating characteristic curve (AUC) to evaluate model outcomes (Bucklin et al., 2015; Zhang et al., 2020). The TSS value ranges from -1 to 1, while the AUC value ranges from 0.5 to 1; a value approaching 1 indicates a stronger predictive capability of the model (Freitas et al., 2019; Zhao et al., 2021). Based on the computational outcomes of 150 models, this study selected those with an average TSS value exceeding 0.8 and an average AUC value greater than 0.9 to generate the final species distribution layers. Furthermore, this study employed the biomod2 to assess the relative importance of the environmental variables utilized in the modeling process, with the methodological rationale detailed in the publication by Xu et al. (2021). The optimal transition threshold (P, in this study, P is 0.379) was determined using the maximum training sensitivity plus specificity Cloglog threshold (Liu et al., 2013). That is, the area with a probability of species presence equal or greater than P (p ≥ P) is considered a suitability area, and the other is a non-suitability area. Furthermore, the suitable habitats were classified into two grades: areas with 0.379 ≤ P < 0.666 were defined as moderately suitable areas, and those with P ≥ 0.666 were defined as core suitable areas.

#### 2.4.2 Correlation between chemical component contents of *E. senticosus* and environmental factors

To analyze the dynamic distribution pattern of the bioactive chemical component content of *E. senticosus* under climate change and the associated environmental impact process, this study used full-subset regression to construct the optimal linear model of chemical component content and environmental variables. Based on the principle of screening by the spatial distance threshold of sampling points, sampling units with a distance of less than 10 km between two points were excluded (Aiello-Lammens et al., 2015). Ultimately, 35 batches of samples were retained for subsequent modeling and analysis. Specifically, based on the distribution data of field-collected samples, this study extracted multidimensional environmental variables corresponding to each sampling site. These variables were obtained from the same environmental database used in the species distribution modeling process and included climatic, soil, NDVI and human footprint variables. The relationships between environmental factors and the accumulation of chemical constituents in *E. senticosus* remain poorly understood, and no consensus framework for variable selection has yet been established. Therefore, a relatively inclusive strategy was adopted in the chemical composition analysis to retain a broader range of potentially relevant environmental information, particularly for soil variables. This approach helps reduce the risk of overlooking important drivers of quality formation. Subsequently, the current climate and soil variables were classified into four categories, including temperature, precipitation, soil chemical properties, and soil physical properties. To address the limited sample size (n = 35) and multicollinearity, we performed Principal Component Analysis (PCA) for the variables within each category. The first two principal components, which cumulatively explained over 70% of the variance, were retained to represent the multidimensional environmental gradients (**Supplementary Figure 1**).

The first two dimensions of PCA effectively capture the key characteristics of the four categories of environmental factors. Among these, a higher value of the first axis of the PCA for temperature-related climate factors (CT-PC1) reflects a higher annual mean temperature and lower variability in the region; a higher value of the second axis (CT-PC2) for temperature-related climate factors reflects a higher temperature during the growing season in the regional climate. A higher value of the first axis of the PCA for moisture-related climate factors (CM-PC1) reflects greater seasonal differences in precipitation and lower precipitation in the driest and coldest quarters within the region; a higher value of the second axis (CM-PC2) for moisture-related climate factors reflects higher annual precipitation and higher precipitation in the wettest quarter in the region. Regarding soil factors, a higher value of the first axis of the PCA for soil physical properties (SP-PC1) reflects a higher volume percentage of coarse particles, lower bulk density, and lower clay content in the regional soil; a higher value of its second axis (SP-PC2) indicates a higher silt content and lower sand content in the regional soil. A higher value of the first axis of the PCA for soil chemical properties (SC-PC1) indicates that the regional soil has higher nutrient content and cation exchange capacity, among other characteristics; a higher value of its second axis (SC-PC2) reflects higher aluminum saturation and a higher carbon-to-nitrogen ratio in the regional soil (**Supplementary Figure 1**).

To depict the comprehensive spatial gradients of various environmental factors, this study projected the multi-layer environmental raster data onto a unified principal component space based on the above PCA results. Specifically, first, the variable values corresponding to each pixel in the multi-band raster were extracted and a pixel-level data matrix was constructed. Then, the trained PCA model was used to predict this matrix to obtain the score of each pixel on each principal component axis. To maintain spatial consistency, the principal component scores were written into a raster template with the same spatial resolution, projection, and extent as the original raster, thereby generating principal component score rasters with layers such as PC1, PC2, and so on (**Supplementary Figures 2**-**5**). This process achieved the spatial dimensionality reduction expression of multi-dimensional environmental variables and provided a unified comprehensive environmental index for subsequent spatial analysis and modeling. Finally, with the support of the MuMIn package in R, a multiple linear model was constructed, taking the content of a single chemical component as the response variable and NDVI, the human footprint and the dimensionally reduced factors as the explanatory variables. Subsequently, the dredge function was used to select the optimal model based on the AIC value. Based on the regression coefficients of each environmental variable in the optimal model, this study replaces the current climate factor layers with the corresponding layers under future climate scenarios, and then predicts the spatial distribution of the content of each active ingredient of *E. senticosus* in Heilongjiang Province under different climate conditions, so as to clarify the impact of climate change on the geographical pattern of its active ingredients.

#### 2.4.3 Planning of priority protection areas and wild tending areas

To support the conservation of *E. senticosus* under global change, we used Zonation 5.0 (https://zonationteam.github.io/Zonation5/), a widely used spatial conservation prioritization software, to identify priority conservation and wild tending areas. Zonation ranks the landscape through an iterative cell-removal algorithm based on the principle of marginal loss, whereby, at each iteration, the grid cell whose removal causes the smallest reduction in overall conservation value is discarded. This process generates a hierarchical priority map that balances the conservation value of all input features while minimizing cumulative conservation loss (Moilanen et al., 2022). For priority reserve planning, we used 13 raster layers representing suitable habitats of *E. senticosus* in Heilongjiang Province under current and future climate scenarios, masked by the overlapping extent of all 13 layers. All input layers were assigned a weight of 1. This equal weighting was adopted to ensure that the prioritization balanced the needs across all temporal scales, without overemphasizing any particular habitat type. The remaining parameters followed the default configuration, and the warp factor was set to 1 (i.e., removing one grid cell per iteration) to optimize the outcome (Moilanen, 2007). Previous studies have confirmed that protecting 5%-20% of a species’ habitat can achieve over 50% of conservation goals (Yang et al., 2019). Based on these findings, we classified the Zonation output in ArcMap 10.5: the top 5% as high-priority protected areas, 5%-10% as medium-priority, and 10%-20% as low-priority protected areas (Moilanen et al., 2005).

Wild tending, a conservation approach that promotes the recovery of wild populations through artificial planting within their native habitats (Chen et al., 2004), is prohibited within existing nature reserves. Therefore, reserve boundaries were first excluded before identifying potential tending areas. To improve the long-term guidance and precision of wild tending, we further incorporated the spatial distribution layers of individual chemical components of *E. senticosus*. For each chemical component and each time period (current and future), a separate Zonation analysis was conducted using the corresponding chemical component distribution layer together with the suitable habitat layer. The parameter settings were identical to those used for priority reserve planning. The resulting continuous priority maps (0-1) were classified using the Natural Breaks method to delineate high-priority wild tending areas. Finally, these maps were overlaid with the county-level administrative boundary dataset to identify county-scale wild tending hotspots, which served as the basis for recommending priority tending locations.

## 3 Results

### 3.1 Dynamic distribution pattern of the suitable habitat for *E. senticosus* in Heilongjiang province under climate change

Under the current climate scenario, the suitable habitat area of *E. senticosus* is approximately 1356,112 km^2^, mainly distributed in China (46.734%), Russia (37.501%), and partially in Japan (7.044%), North Korea (6.778%), and South Korea (1.944%) (**Figures 2A, B**). This distribution pattern is mainly regulated by environmental factors such as Bio18, Bio11, NDVI and Bio08 (**Supplementary Table 3**). Under the current climate scenario, China has the largest general and core suitable areas for *E. senticosus*, which are approximately 270,528 km^2^ and 363,234 km^2^ respectively (**Figure 2B**). Within the territory of China, Heilongjiang Province has the largest area of both types of suitable areas. The area of its general suitable areas accounts for 37.155% of the total area of the same type of suitable areas in China, and the area of its core suitable areas even reaches 59.547% (**Figure 2C**). From a spatial perspective, the suitable habitat for *E. senticosus* within Heilongjiang Province is mainly distributed in the forests and the forest-grassland ecotone (**Figure 2D**). Among them, the general suitable areas are distributed in the junctions between the western grassland vegetation zone (Songnen Plain) and the central forest vegetation zones (Lesser Khingan Mountains, etc.) of Heilongjiang Province, and the other part is distributed in the Sanjiang Plain (**Figures 1B**, **2D**). The core suitable habitat is extensively distributed in forest vegetation zones such as the Lesser Khingan Mountains, Wanda Mountains, and the northern branches of the Changbai Mountains in central and southeastern Heilongjiang Province.

**Figure 2:**
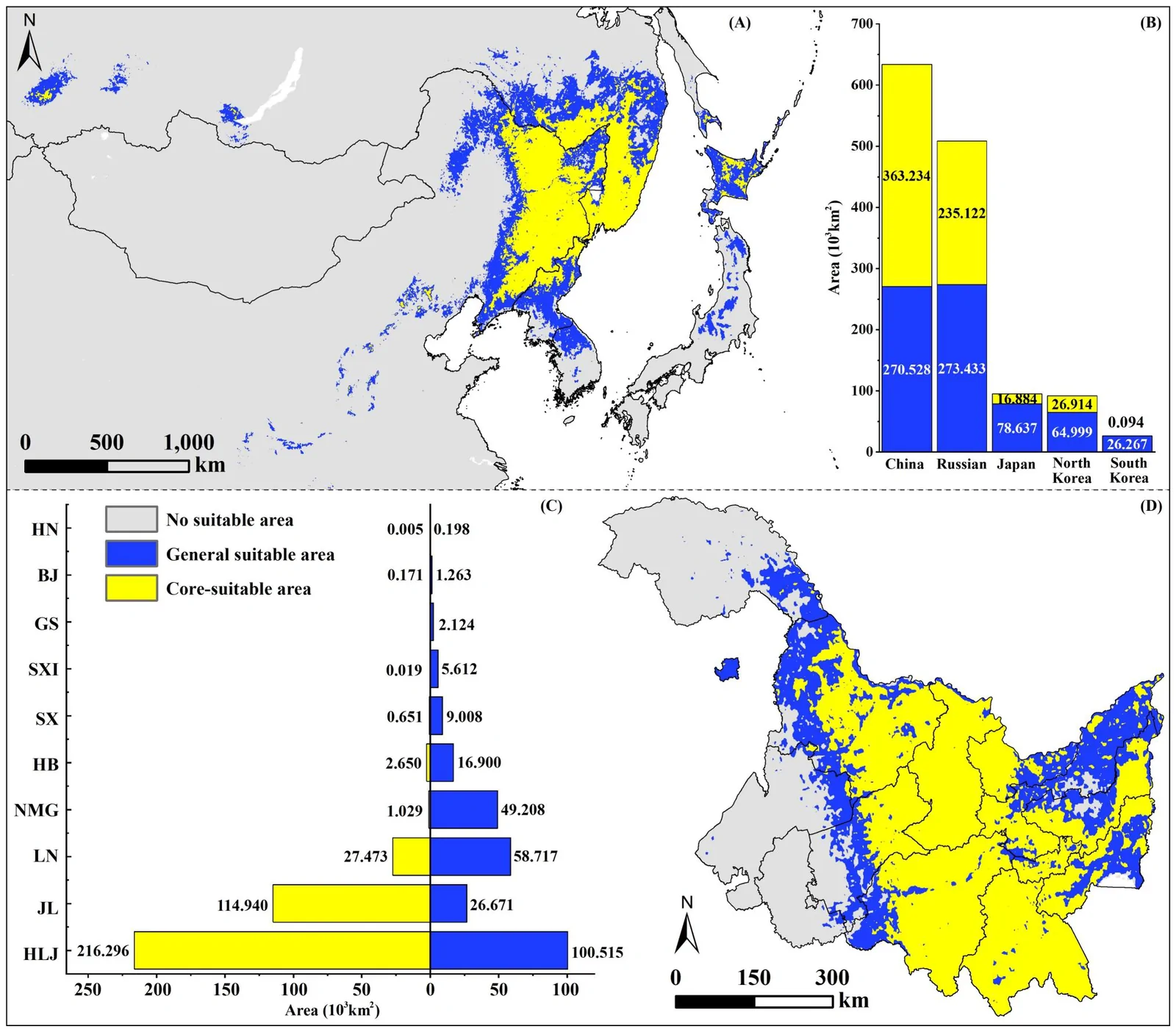
Distribution patterns of the suitable habitat for *E. senticosus* simulated by the species distribution model. **(A)** Distribution pattern of the suitable habitat under the complete ecological niche; **(B)** Area of suitable habitat distributed across different countries; **(C)** The top 10 provinces in China ranked by total suitable habitat area of *E. senticosus are* as follows: HLJ, Heilongjiang Province; JL, Jilin Province; LN, Liaoning Province; NMG, Inner Mongolia Autonomous Region; HB, Hebei Province; SX, Shanxi Province; SXI, Shaanxi Province; GS, Gansu Province; BJ, Beijing City; HN, Henan Province; **(D)** Spatial distribution pattern of the suitable habitat in Heilongjiang Province.

In the future, the total suitable area for *E. senticosus* in Heilongjiang Province will generally show an upward trend, but it will exhibit significant differences under different carbon emission scenarios (**Figures 3A, B**). Under the low-medium carbon emission scenario (SSP126, SSP245, SSP370), the total suitable habitat area of *E. senticosus* initially increases and then decreases with the passage of years. In contrast, under the high carbon emission scenarios (SSP585), the total suitable habitat area generally exhibits a shrinking trend over time, and only under the 2090s-SSP585 climate scenario does the total suitable habitat area decrease compared with the current period (**Figure 3B**). From a spatial perspective, climate change will not cause a significant spatial migration of the total suitable habitat for *E. senticosus* in Heilongjiang Province (**Figure 3A**). However, with the rise in carbon emission concentration and the passage of time, climate change is expected to severely damage the core suitable habitat of *E. senticosus* in Heilongjiang Province. By the end of the century, more than 99% of the core suitable habitats are expected to be lost under higher-emission scenarios. Only a small remnant of core suitable habitat will remain in the western part of the Lesser Khingan Mountains, whereas the remaining core habitats are anticipated to experience severe contraction. The dominant climate factors (Bio18, Bio11 and Bio08) influencing the distribution of *E. senticosus* all show an upward trend with increasing carbon emission concentration and time. Under the 2090s-SSP585 scenario, the increments are the largest. Specifically, Bio18 (warmest quarter precipitation) increases to 401.359 mm, Bio11 (coldest quarter mean temperature) to -10.237 °C, and Bio08 (wettest quarter mean temperature) to 26.822 °C (**Supplementary Table 4**).

**Figure 3:**
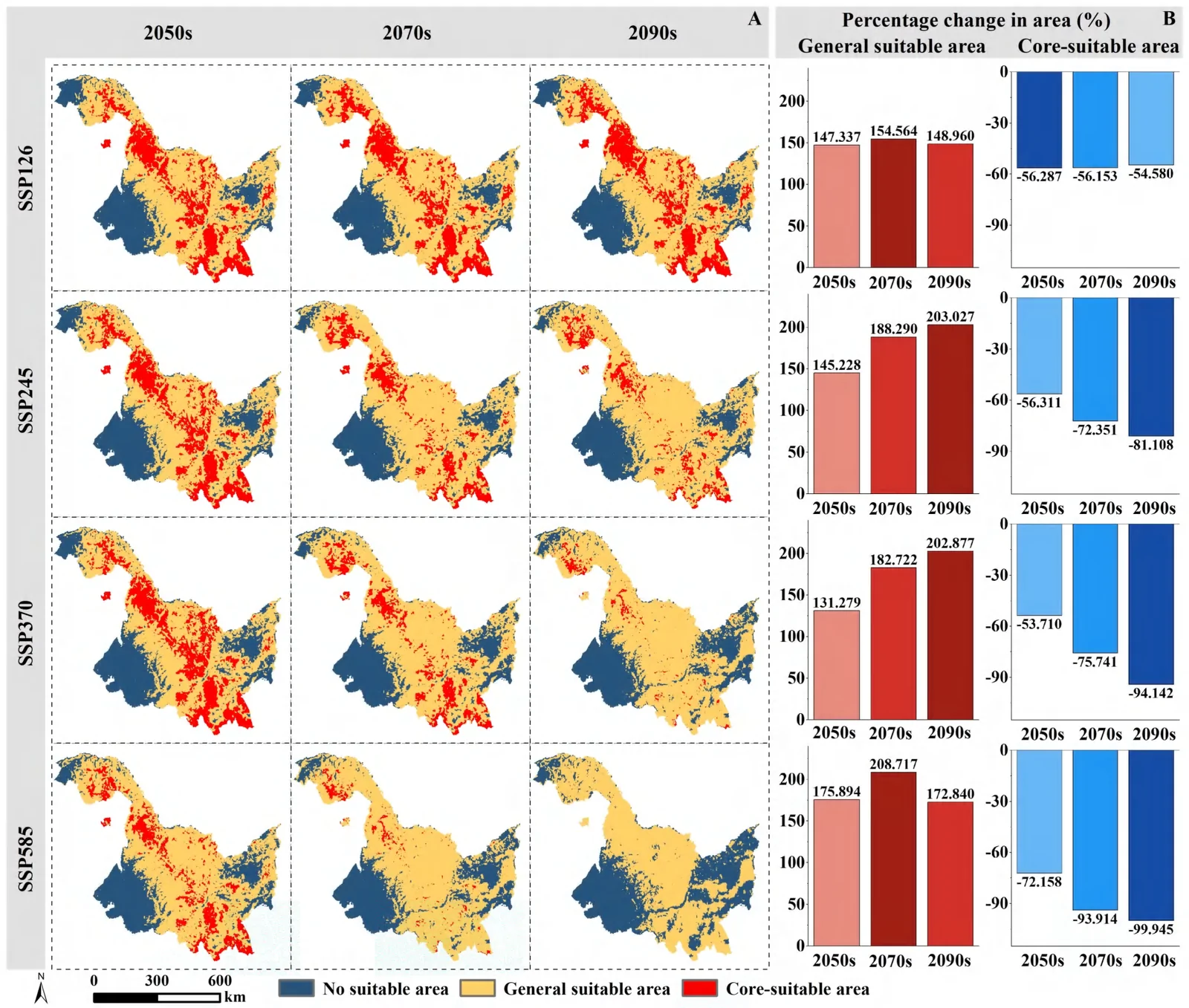
Distribution patterns of suitable habitat for *E. senticosus* in Heilongjiang Province under different future climate scenarios. **(A)** Changes in spatial distribution patterns; **(B)** Changes in area of different suitability grades. SSP126, Low concentration carbon emissions; SSP245, Medium concentration carbon emissions; SSP370, Medium-high concentration carbon emissions; SSP585: High concentration carbon emissions.

### 3.2 Changes in habitat area of *E. senticosus* in Heilongjiang Province under land use changes

Under the current climatic conditions, the total suitable area for *E. senticosus* in Heilongjiang Province is approximately 113,711 km^2^ of forest land. Under the premise that the population of *E. senticosus* lags behind in its movement to suitable climate areas (Supposition 1), the total area of its habitat (forest) is expected to increase in the overlapping regions of different suitable climate areas and the current one (**Figure 4**). Among these, a sustained growth trend is observed under the SSP245 and SSP370 scenarios; under the SSP126 and SSP585 climate scenarios, a trend of first increasing and then decreasing is exhibited. Under the premise that the *E. senticosus* population can migrate in response to climate change (Supposition 2), the simulated habitat area within the suitable range of *E. senticosus* under different climate scenario combinations will still be higher than the current suitable habitat area (**Figure 4**). Compared with the current period, a trend of first increasing and then decreasing is observed under the SSP126 and SSP585 scenario; under the SSP245 and SSP370 scenarios, a sustained growth trend is exhibited, with the maximum increase occurring in the 2090s-SSP370 combination, which is approximately 54,888 km^2^ higher than the current value.

**Figure 4:**
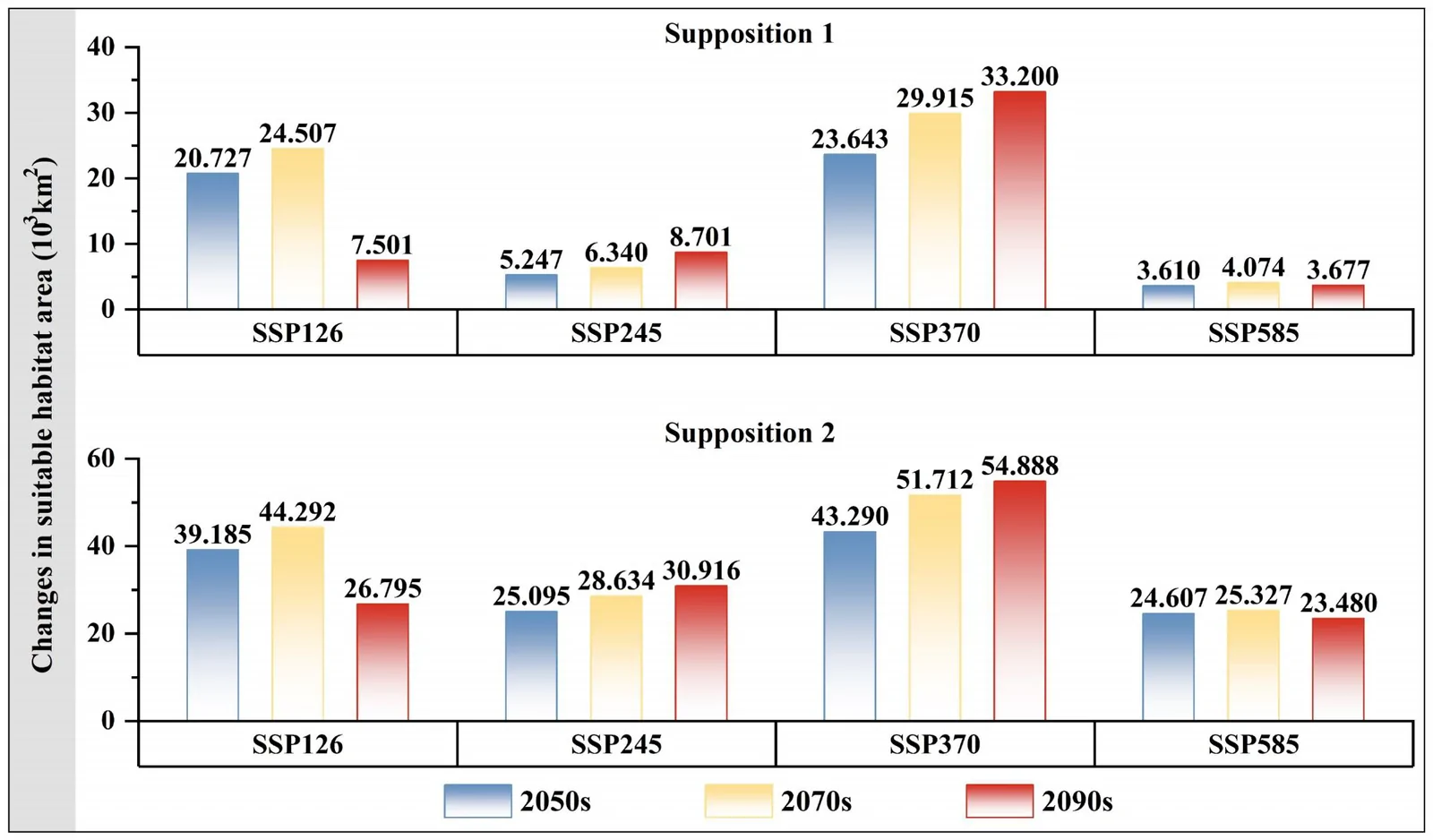
Impact of future land-use change on the habitat of *E. senticosus* in Heilongjiang Province.

### 3.3 Content, spatial distribution characteristics of active chemical components of *E. senticosus* and their responses to climate change

The fingerprinting results (**Figure 5**) revealed the distribution characteristics of key bioactive components among the tested samples, with specific samples exhibiting relatively high contents of different target components. The contents of each component vary due to the influence of environmental factors such as soil and climate (**Table 2**). Among the various chemical components, the contents of comprehensive chemical components (CC-PCA), coniferin, syringin, eleutheroside E, isofraxidin, and syringaresinol-4’-O-*β*-D-glucopyranoside exhibit significant optimal association models with multi-dimensional environmental variables. In particular, the content of comprehensive chemical components is mainly regulated by climatic factors, and its optimal variable set includes CM-PC1, CM-PC2, and CT-PC1. The optimal set of environmental factors for predicting the content of coniferin consists of CM-PC2, CT-PC2, and SP-PC1. CM-PC2 constitute the optimal variable set for predicting variations in the content of syringin. The content of eleutheroside E is influenced by NDVI. The optimal variable for predicting variations in the content of isofraxidin is CM-PC1. The optimal variable set for predicting syringaresinol-4’-O-*β*-D-glucopyranoside consists of CM-PC2 and SP-PC2.

**Figure 5:**
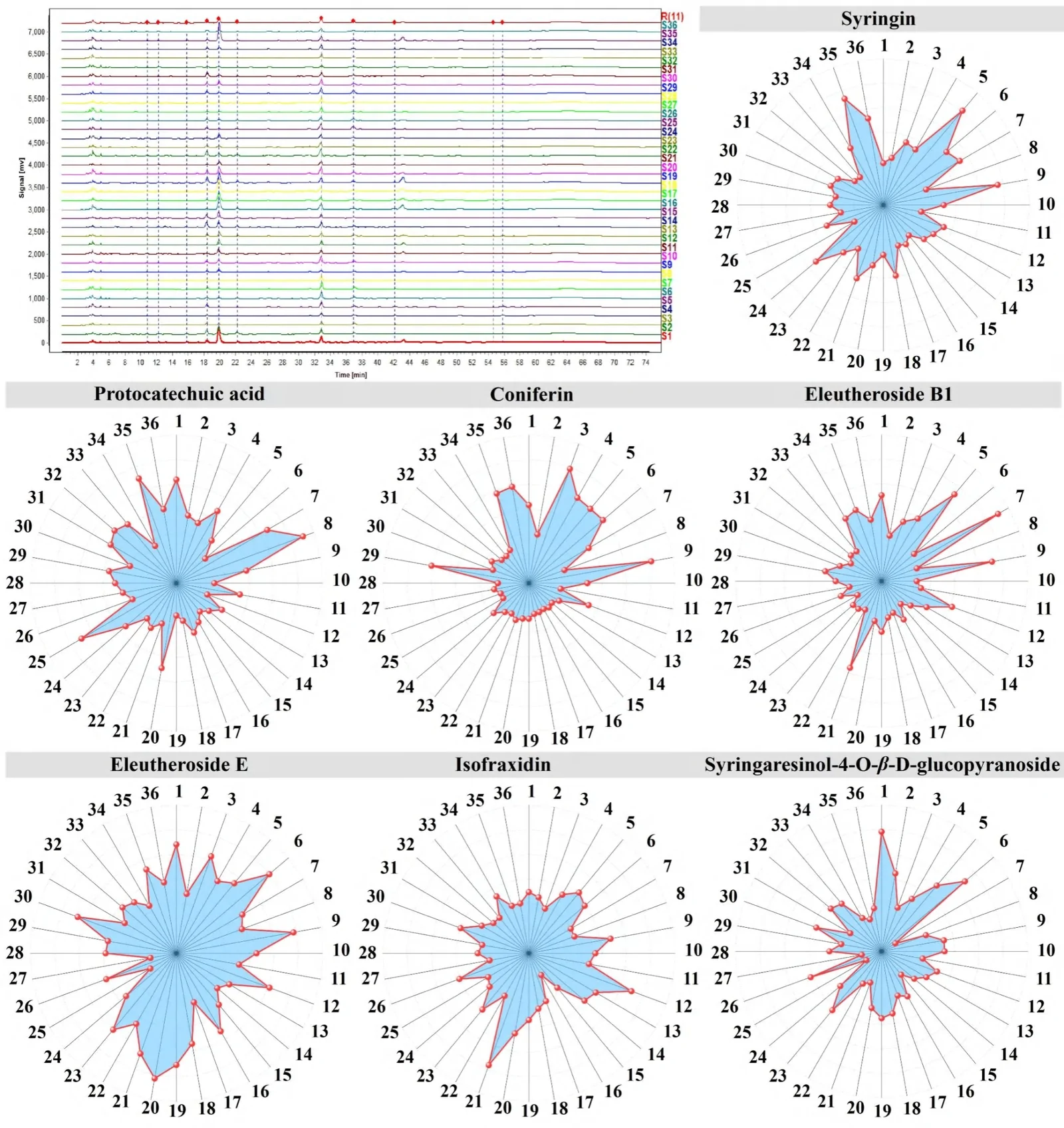
Fingerprint chromatogram and content comparison of 36 batches of *E.senticosus* samples. The radar chart is solely intended for relative comparison of content across samples and does not denote specific quantitative values.

**Table 2:** Optimal prediction models for the contents of 6 chemical components.

Response variable	Optimal linear model	*R* ^2adj^	*p*-value
*C_CC-_*_PCA_	*C_CC-_*_PCA_ = -0.1133×CM-PC1 + 0.1747×CM-PC2 - 0.1146×CT-PC1	0.2980	0.002848**
*C*_coniferin_	*C*_coniferin_= 0.2151×CM-PC2 + 0.2067×CT-PC2 - 0.2406×SP-PC1	0.2838	0.003825**
*C*_syringin_	*C*_syringin_ = 0.2253×CM-PC2	0.1329	0.01791*
*C*_eleutheroside E_	*C*_eleutheroside E_ = 1.6908×NDVI	0.1032	0.03365*
*C*_isofraxidin_	*C*_isofraxidin_ = -0.2126×CM-PC1	0.1766	0.006942**
*C*_syringaresinol-4’-O-_ *_β_*_-D-glucopyranoside_	*C*_syringaresinol-4’-O-_*_β_*_-D-glucopyranoside_ = 0.2478×CM-PC2 - 0.234×SP-PC2	0.1633	0.02188*

(*: p < 0.05; **: p < 0.01.).

From a spatial perspective, the regions with high contents of comprehensive chemical components are mainly distributed in the Lesser Khingan Mountains and Wanda Mountains in Heilongjiang Province; the regions with high coniferin content are mainly distributed in the forest-grass transition zones in the eastern Songnen Plain and the Sanjiang Plain; the high-content distribution pattern of syringin is similar to that of syringaresinol-4’-O-*β*-D-glucopyranoside and eleutheroside E, and is mainly concentrated in the Lesser Khingan Mountains, the Wanda Mountains and a small area in the northern part of the Changbai Mountains; the high-content distribution area of isofraxidin is concentrated in the Wanda Mountains and the eastern part of the Sanjiang Plain (**Figure 6A**).

**Figure 6:**
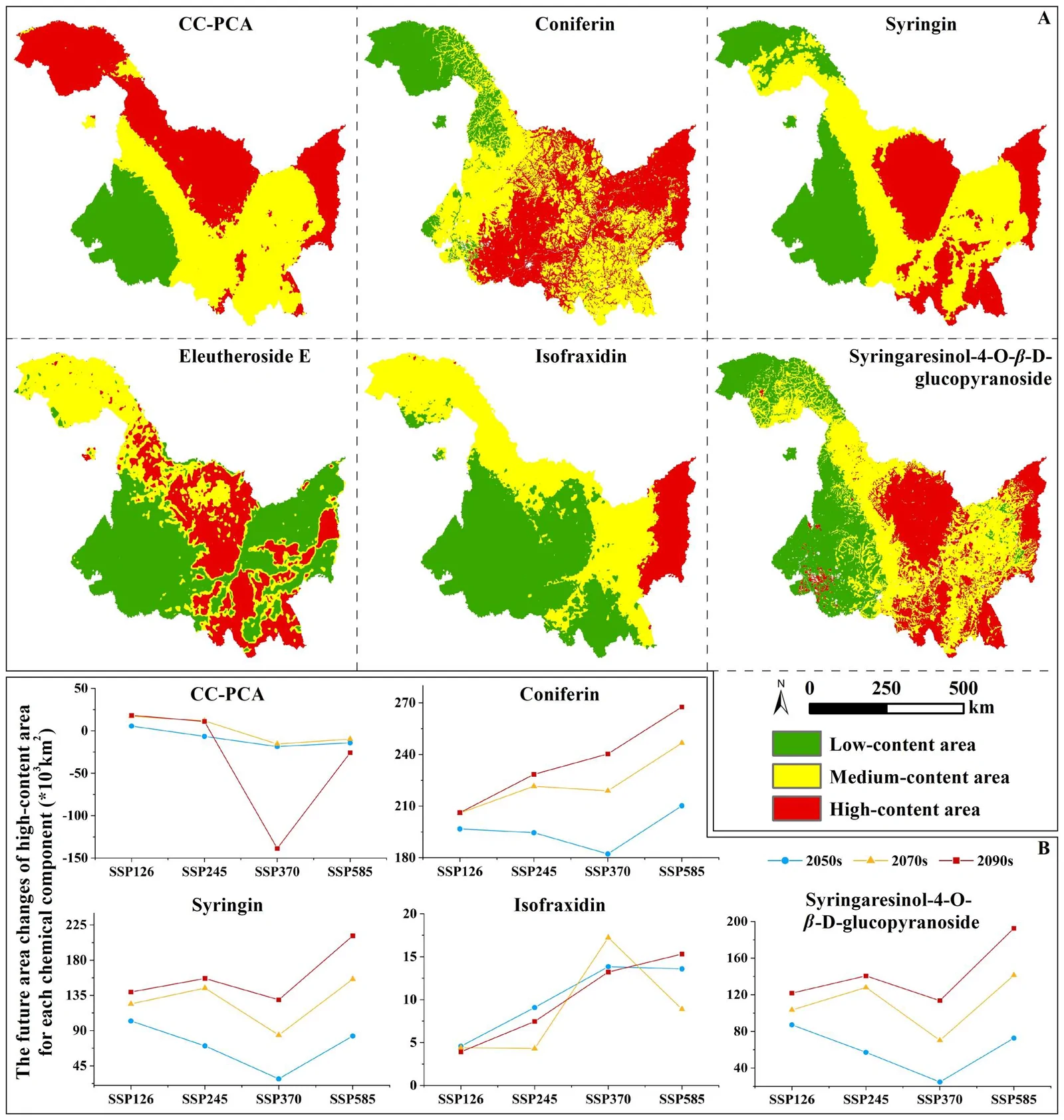
Changes in the effective chemical components of *E. senticosus* in Heilongjiang Province under current and future conditions. **(A)** Spatial distribution of each chemical component of *E. senticosus* in Heilongjiang Province under current climate conditions; **(B)** Changes in the area of high-content regions for each chemical component of *E. senticosus* in Heilongjiang Province under future climate change.

Under future climate change scenarios, no significant spatial alterations were observed in the distribution of these components (**Supplementary Figures S6**-**S10**). From an areal perspective, the areas of high-content distribution regions for all components other than comprehensive chemical components show an increasing trend (**Figure 6B**). Specifically, the distribution areas of low and medium contents for three chemical components coniferin, syringin, and syringaresinol-4’-O-*β*-D-glucopyranoside will decrease sharply with the increase in years and carbon emissions, while the distribution areas of high contents will increase sharply. The high-content distribution areas of isofraxidin also showed an increasing trend, but the growth rates was relatively slow. Overall, the high-content distribution areas of the comprehensive chemical components exhibited an expanding trend under the low-carbon emission scenario, while a shrinking trend was observed under the medium and high-carbon emission scenarios.

### 3.4 Protected areas and wild tending areas of *E. senticosus* in Heilongjiang province under climate and land use change

Against the backdrop of global change in this century, the total area of potential protected areas for *E. senticosus* in Heilongjiang Province is approximately 18,653 km^2^, accounting for 16.404% of the current suitable habitat area of the species. Among these, the areas of high-priority and medium-priority protected areas are both approximately 4,663 km^2^, while the area of low-priority protected areas is about 9,327 km^2^ (**Figure 7A**). Within the potential protected areas of *E. senticosus*, the existing nature reserves cover an area of approximately 2,406 km^2^, accounting for 12.899% of the total potential protected area. Within the existing nature reserves, the low-priority protected area had the largest coverage, approximately 1,184 km^2^ accounting for 12.694% of the identified low-priority protected area; followed by the high-priority protected area, with a coverage of about 738 km^2^; and the medium-priority protected area had the smallest coverage, approximately 484 km^2^. At the individual protected site scale, Mudanjiang Jingpo Lake Scenic Area harbored the largest total area of *E. senticosus*-targeted potential protected areas, as well as the largest areas of low-priority potential protected areas; in contrast, Shengshan National Nature Reserve was the protected site with the largest area of high and medium-priority potential protected areas.

**Figure 7:**
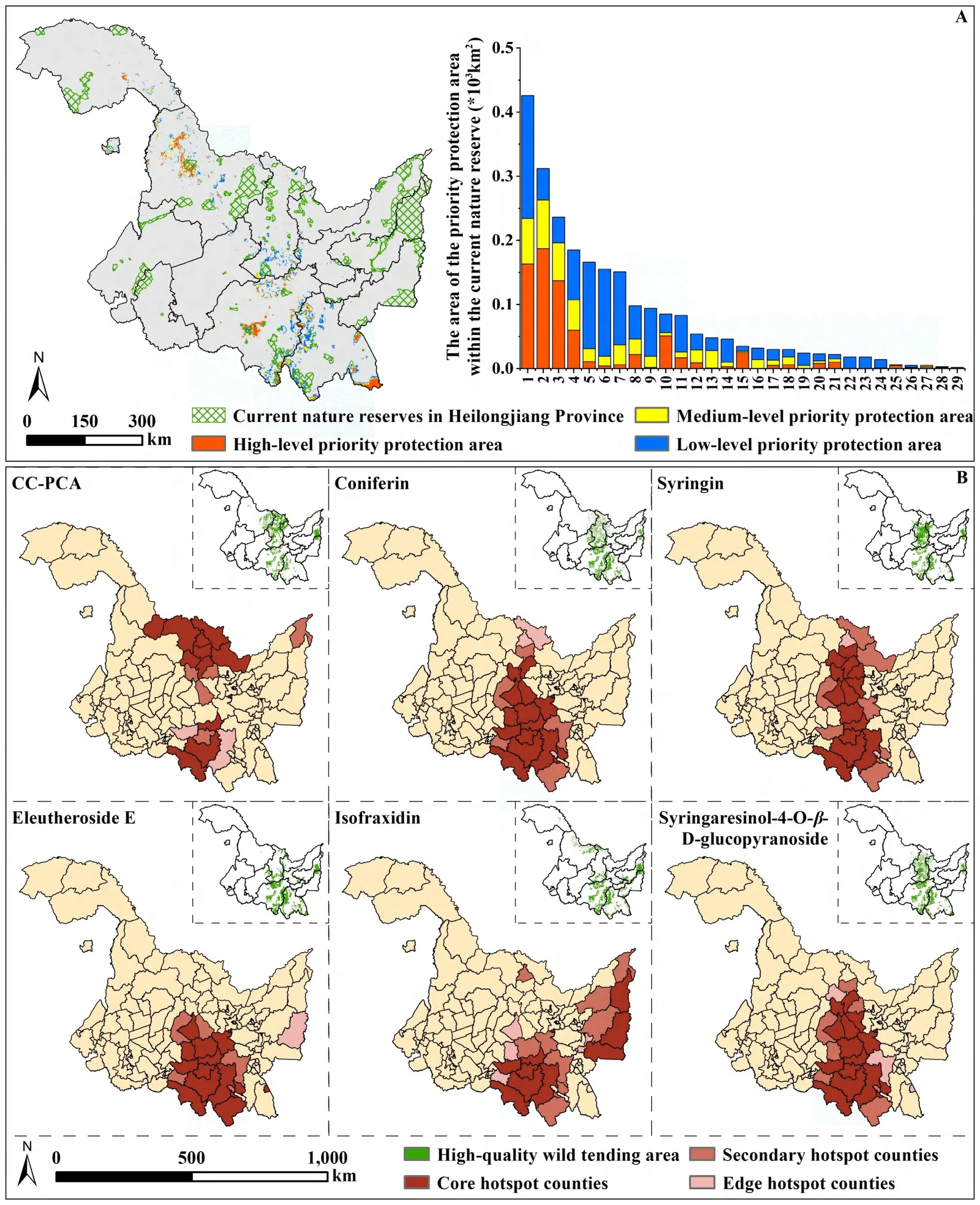
Protective areas of *E. senticosus* in Heilongjiang Province and the zonation of its wild tending areas under current climatic conditions. **(A)** Priority protection areas for *E. senticosus* in Heilongjiang Province within this century: the correspondence between the protection zone serial numbers and the corresponding information is presented in **Supplementary Table 5**; **(B)** County-level planning of comprehensive and directional wild tending zones for *E. senticosus* in Heilongjiang Province under current climatic conditions.

Under the current climate, the Lesser Khingan Mountains, Zhangguangcai Range and Wanda Mountain ranges are the main areas suitable for the construction of wild tending areas (**Figure 7B**). Specifically, when targeting high-quality comprehensive chemical component content, counties and districts in northern Yichun and eastern Harbin are identified as priority areas for the wild tending of *E. senticosus*. When targeting high-quality coniferin and syringin content, counties and districts in southern Yichun and eastern Harbin are priority areas for the wild tending of *E. senticosus*. When targeting high-quality eleutheroside E content, border counties and districts in western Mudanjiang and eastern Harbin are priority areas for the wild tending of *E. senticosus*. When targeting high-quality isofraxidin content, western Mudanjiang, southeastern Harbin, northeastern Jixi and eastern Shuangyashan are priority areas for the wild tending of *E. senticosus*. When targeting high-quality syringaresinol-4’-O-*β*-D-glucopyranoside content, counties and districts in southern Yichun, eastern Harbin and western Mudanjiang are priority areas for the wild tending of *E. senticosus.*

Under future climate conditions, the significant hotspot counties suitable for wild tending activities targeting comprehensive chemical components, coniferin, syringin and syringaresinol-4’-O-*β*-D-glucopyranoside exhibit a distinct northwestward shift trend, primarily transferring toward Heihe City and the Greater Khingan Mountains region (**Supplementary Figures S11**-**S15**). Similarly, with the exception of some districts and counties in the Wanda Mountains region, the significant hotspot counties currently suitable for wild tending activities targeting isofraxidin will almost disappear; instead, their hotspot areas will shift northwestward and be replaced by counties under the jurisdiction of the Greater Khingan Mountains region and Heihe City.

## 4 Discussion

### 4.1 Methodological improvements for potential suitable area prediction

The distribution pattern of *E. senticosus* is primarily influenced by the combined effects of climatic hydrothermal conditions (**Supplementary Table 3**). Among these factors, the precipitation of Bio18 is the key determinant of its potential suitable range, followed by Bio11; meanwhile, the NDVI, and Bio08, collectively influence its ecological suitability to a certain extent (**Supplementary Table 3**). This result is generally consistent with the conclusions of existing studies (Wang et al., 2019; Lin et al., 2024), but there are certain differences in the specific dominant factors, which may be attributed to variations in modeling scope, distribution data, and model algorithms. First, differences in modeling scope are one of the key reasons. Most existing studies are based on regional models, which only reflect partial niche characteristics of *E. senticosus* in Northeast China (Wang et al., 2019; Lin et al., 2024). In contrast, this study constructed a full-niche model across a broader geographic scope (Asian scale), which enables the integration of more comprehensive environmental gradients and distribution characteristics (Pearson et al., 2004). However, incorporating regions that are potentially suitable but inaccessible to the natural dispersal of the species into the model may lead to a decrease in prediction accuracy (Acevedo et al., 2012). Thus, this study refers to the perspective of Barve et al. (2011) and defines the accessibility boundary based on the historical distribution records of *E. senticosus*, achieving a balance between model representativeness and ecological realism. Second, the completeness of distribution data is another key source of differences. Based on conventional databases, this study further used the plantR package for quality control and supplementary screening of occurrence records, thereby obtaining more complete presence records. This data advantage enhances the spatial coverage and prediction stability of the model, making the results more representative at the regional scale. Finally, differences in model algorithms also contribute to the observed variations. Previous studies mostly adopted a single algorithm (MaxEnt). In contrast, this study used an optimized ensemble model Biomod2, which integrates multiple algorithms to reduce the instability and systematic bias of single models (Araujo and New, 2007; Goldsmit et al., 2020). This ensemble model more accurately characterizes the dynamic changes in suitable areas of *E. senticosus* under different climate scenarios.

It should be noted that this study assumed that these environmental variables remained constant under future climate scenarios. While this assumption enables the assessment of climate-driven changes in habitat suitability, it may not fully capture potential shifts caused by future changes in soil processes, vegetation productivity, and human activities. Future studies integrating dynamic projections of multiple environmental components will help improve the accuracy and ecological realism of long-term habitat suitability assessments. In addition, incorporating depth-specific soil information may further improve the ecological interpretation of species-environment relationships in future studies.

### 4.2 Analysis of the environmental driving mechanism of *E. senticosus* distribution

The Biomod2 predictions indicate that *E. senticosus* is primarily distributed in the temperate region of Northeast Asia, with core suitable habitats concentrated in Northeast China and the Russian Far East. Within China, Heilongjiang Province contains the largest areas of both general and core suitable habitats, mainly distributed across the Lesser Khingan Mountains, Wanda Mountains, and the northern margin of the Changbai Mountains (**Figures 1**, **2**). These regions are characterized by cool and humid climates and extensive mixed forests, which are consistent with the ecological preferences of *E. senticosus* (Lin et al., 2024). Among all environmental variables, Bio18 (precipitation of the warmest quarter) was identified as the most important predictor (**Supplementary Table 3**). Water availability during the growing season directly influences plant growth, regeneration, and population persistence (Knapp et al., 2002; Huxman et al., 2004). Adequate summer precipitation can maintain favorable soil moisture conditions and support photosynthetic activity, whereas prolonged drought may restrict root water uptake and reduce carbon assimilation (Brunner et al., 2015; Seleiman et al., 2021). Conversely, excessive precipitation may negatively affect flowering and reproductive success, particularly during the flowering and fruiting periods of *E. senticosus* (Huang, 2017). These findings suggest that the species is sensitive to both water limitation and excessive moisture, highlighting the critical role of precipitation in shaping its distribution.

Bio11 (mean temperature of the coldest quarter) was the second most influential variable (**Supplementary Table 3**). Winter temperature can affect overwinter survival, root freezing risk, and spring phenology (Repo et al., 2014; Ambroise et al., 2020; Ritonga and Chen, 2020). Extremely low winter temperatures may shorten the effective growing season by delaying spring growth and increasing physiological stress (Inouye, 2008). This may partly explain the absence of natural populations in the colder Greater Khingan Range region of Heilongjiang Province. NDVI also contributed substantially to habitat suitability. As an indicator of vegetation productivity and coverage, NDVI reflects local resource availability and competitive conditions. Moderate vegetation cover may improve habitat quality through enhanced soil fertility and moisture retention, whereas excessive canopy closure can reduce understory light availability and intensify competition for resources, thereby limiting population regeneration (Chen et al., 1999; Six et al., 2000; Valladares and Niinemets, 2008). In addition, Bio08 may further influence habitat suitability by regulating temperature and moisture conditions during critical growth periods.

### 4.3 Dynamics and survival risks of *E. senticosus* habitats in Heilongjiang Province under future climate scenarios

Future species persistence is jointly determined by changes in climate suitability, land-use dynamics, and species-specific ecological traits (Jump and Peñuelas, 2005; Guisan et al., 2013). For *E. senticosus* in Heilongjiang Province, the interaction among these factors leads to contrasting ecological outcomes under different climate scenarios. Under the low-emission scenario (SSP126), relatively stable hydrothermal conditions support the persistence of climatically suitable habitats despite a slight reduction in core suitable areas (**Supplementary Table 4**; **Figure 3**). Meanwhile, the expansion of forest land provides additional habitat resources (**Supplementary Figure 4**), allowing populations to maintain viability even under dispersal limitations (Xu et al., 2018; Wijenayake et al., 2025). When migration is possible (Supposition 2), populations can further colonize newly suitable areas, while gene flow between core and marginal populations may enhance adaptive capacity and long-term persistence (Aitken et al., 2008; Sexton et al., 2009).

In contrast, from SSP245 onward, core climate-suitable areas show a continuous decline despite ongoing forest expansion (**Figure 3**). This suggests that newly added forest land cannot compensate for the ecological functions provided by high-quality core habitats. The loss of core areas may reduce reproductive sources, constrain recruitment, and increase population vulnerability (Li et al., 2024). These risks become particularly pronounced under SSP585. Although forest area generally continues to increase, core climate-suitable habitats are projected to decline by more than 99% by the end of the century (**Figure 3**). Most newly formed habitats are marginally suitable and cannot provide environmental conditions comparable to those of the current core areas (Chen et al., 2011). Under migration lag (Supposition 1), populations may remain trapped in increasingly unsuitable habitats, facing elevated risks of heat stress, drought, and eventual local extinction (Aitken et al., 2008). Even under migration (Supposition 2), successful establishment in newly colonized areas may be limited by immature forest structures and unstable understory environments (Anderegg et al., 2013). Therefore, climate-driven loss of core suitable habitats, rather than forest area reduction, is likely to be the primary threat to the long-term persistence of *E. senticosus* in Heilongjiang Province.

### 4.4 Changes in chemical component contents imply opportunities and challenges for the conservation and utilization of wild *E. senticosus* resources in Heilongjiang province under climate change

The major bioactive constituents of *E. senticosus*, including syringin, syringaresinol-4′-O-*β*-D-glucopyranoside, coniferin, isofraxidin, and eleutheroside E, all originate from the phenylpropanoid pathway, yet their accumulation exhibited distinct responses to environmental gradients. These differences likely reflect divergent downstream metabolic regulation despite their shared biosynthetic origin (Vogt, 2010). Among the environmental factors examined, moisture-related climatic conditions emerged as the predominant predictors of chemical composition (**Table 1**). The moisture gradient represented by CM-PC2, which reflects higher annual precipitation and greater precipitation during the wettest quarter, was positively associated with the accumulation of coniferin, syringin, and syringaresinol-4′-O-*β*-D-glucopyranoside, and also contributed positively to the integrated chemical composition (*C_CC-_*_PCA_). Conversely, CM-PC1, representing greater precipitation seasonality together with lower precipitation during the driest and coldest quarters, showed negative associations with *C_CC-_*_PCA_ and isofraxidin. Collectively, these relationships indicate that relatively humid environments with stable precipitation regimes favor the accumulation of multiple phenylpropanoid-derived metabolites. This pattern is consistent with previous studies showing that adequate water availability generally supports photosynthetic carbon assimilation, maintains metabolic activity, and facilitates carbon allocation into secondary metabolism (Vincent et al., 2005; Anjum et al., 2011; Wang et al., 2020; Tanbakuyi et al., 2025). Stable moisture conditions may therefore provide a favorable physiological environment for phenylpropanoid biosynthesis, whereas increased precipitation seasonality could impose intermittent water stress that constrains metabolite accumulation.

Temperature-related variables influenced a smaller subset of compounds (**Table 1**). Coniferin was positively associated with CT-PC2, indicating higher concentrations under climates characterized by warmer growing seasons, whereas *C_CC-_*_PCA_ was negatively associated with CT-PC1, suggesting that lower annual mean temperature together with greater thermal variability may favor the overall accumulation of bioactive compounds. Because coniferin serves as a glycosylated storage form of monolignols (Lanot et al., 2006; Speeckaert et al., 2022), warmer growing-season temperatures may increase carbon assimilation and carbohydrate availability, thereby supporting glycosylation and metabolite storage (Dixon and Barros, 2019). However, the contrasting response of *C_CC-_*_PCA_ also suggests that the effects of temperature differ among metabolites, highlighting that different branches of the phenylpropanoid pathway may respond differently to climatic warming.

Soil physical properties exerted compound-specific influences on metabolite accumulation (**Table 1**). Coniferin was negatively associated with SP-PC1, indicating higher concentrations in relatively fine-textured soils characterized by higher clay content and lower proportions of coarse particles. Such soils generally retain water more effectively and provide more stable moisture conditions for root growth and physiological activity (Hillel, 2003), potentially supporting sustained precursor production for secondary metabolism. In contrast, syringaresinol-4′-O-*β*-D-glucopyranoside showed a negative association with SP-PC2, suggesting relatively higher concentrations in sandier soils with lower silt content. These contrasting responses imply that although water availability represents a common environmental driver, individual metabolites exhibit distinct sensitivities to soil texture, possibly reflecting differences in carbon allocation, transport, or storage among downstream branches of the phenylpropanoid pathway.

Compared with climatic and soil variables, vegetation conditions showed a more limited influence on chemical composition (**Table 1**). NDVI was retained only in the optimal model for eleutheroside E, indicating that this compound is more strongly associated with vegetation structure than with the broader climatic gradients captured by the PCA axes. Higher NDVI generally reflects denser vegetation cover and more stable understory microclimatic conditions, including moderated light environments and improved soil moisture retention (Yang et al., 2023). Such habitats may provide relatively stable physiological conditions that favor carbon assimilation and sustain the production of specific phenylpropanoid-derived metabolites.

It is noteworthy that this study provides valuable insights into the potential impacts of climate change on the habitat suitability and medicinal quality of *E. senticosus* in Heilongjiang Province, the applicability of these findings should be interpreted within the regional context. The bioactive compound models developed in this study were based on samples collected across Heilongjiang Province and therefore provide meaningful predictions for resource conservation and utilization within this region. However, their application to broader geographic scales requires further validation using larger-scale sampling and additional environmental gradients. Furthermore, the predicted responses of bioactive compounds under future climate scenarios were based on the current relationships between climatic factors and metabolite accumulation. Future studies integrating large-scale chemical sampling, long-term field observations, and controlled experiments (e.g., warming and precipitation manipulation) will help validate model predictions and further clarify the mechanisms underlying climate-driven changes in the medicinal quality of *E. senticosus*.

### 4.5 Implications for conservation and wild tending under future climate change

Future climate change poses different challenges to the conservation and sustainable utilization of *E. senticosus*, indicating that conservation and wild tending should be planned using different management objectives rather than a single habitat suitability criterion.

From a conservation perspective, although the total suitable habitat of *E. senticosus* is projected to remain relatively stable under most future climate scenarios, the marked contraction of climatically stable core habitats under high-emission scenarios suggests that future conservation should focus on maintaining long-term population persistence rather than simply maximizing the total suitable area. Our results further reveal that only 12.899% of the identified priority conservation areas are currently covered by existing nature reserves (**Figure 7A**), indicating substantial conservation gaps. Therefore, future conservation efforts should prioritize climatically stable refugia, particularly in the western Lesser Khingan Mountains (**Figures 1B**, **7**), while strengthening protection in currently unprotected priority areas through the establishment of provincial germplasm conservation sites and long-term ecological monitoring programs.

From a wild tending perspective, habitat suitability alone is insufficient for determining suitable tending locations because medicinal quality is regulated by environmental drivers that differ from those controlling species distribution. Consequently, regions suitable for conserving wild populations do not necessarily coincide with areas that maximize medicinal quality (**Figures 3**, **6**). Targeted wild tending should therefore be implemented according to specific medicinal objectives. For example, areas in southern Yichun are more suitable for producing coniferin- and syringin-rich medicinal materials (**Figure 7B**), whereas other hotspot regions should be selected according to the desired active compounds. Such differentiated management strategies could improve both the quality of medicinal materials and the long-term sustainability of wild resource utilization.

## 5 Conclusion

This study combining ensemble species distribution models, HPLC fingerprint-based medicinal quality assessment, and spatial conservation prioritization to evaluate the impacts of climate change on the habitat suitability, bioactive compound distribution of *E. senticosus*. Our results demonstrated that the distribution of *E. senticosus* was mainly controlled by hydrothermal conditions, with Bio18, Bio11, and Bio08 identified as the dominant climatic drivers. Although future climate change may expand the overall suitable habitat area in Heilongjiang Province, the pronounced contraction of core suitable habitats under high-emission scenarios suggests potential risks to long-term population persistence. The accumulation patterns of major bioactive compounds exhibited distinct environmental responses, indicating that medicinal quality does not necessarily coincide with habitat suitability. Spatial conservation prioritization identified approximately 18,653 km^2^ as potential conservation areas, whereas existing nature reserves covered only 12.899% of these priority areas, highlighting considerable conservation gaps. Wild tending priority areas were mainly concentrated in the Lesser Khingan Mountains under current conditions and showed a northwestward shift toward the western Lesser Khingan Mountains and Greater Khingan Mountains region under future climate scenarios. Overall, this study highlights the importance of integrating ecological suitability, medicinal quality, and conservation planning to improve the adaptive management of wild medicinal plant resources under global change.

## Data Availability

The datasets presented in this study can be found in online repositories. The names of the repository/repositories and accession number(s) can be found below: https://zenodo.org/records/19230896.
