# Supplementary material for: Habitat suitability and active component responses of Eleutherococcus senticosus under climate change: implications for conservation and wild tending
Source: Front Plant Sci. 2026 Sep 14;17:1871650. doi: 10.3389/fpls.2026.1871650 (PMC13616800; doi:10.3389/fpls.2026.1871650)
Supplement: Supplementary file 1 [file Table1.docx]

**Supporting Information**

**Supporting Text**

**A Optimize the MaxEnt model**

To optimize the MaxEnt model, the present study employed the kuenm package in R to evaluate model performance under various combinations of regularization multipliers (RM) and feature classes (FC) (Cobos *et al.*, 2019). To optimize the model by combining 17 regularization multiplier values (0.1–1 at 0.1 intervals; 2–6 at 1 interval; and 8, 10) with the five features offered by the MaxEnt model, namely, linear features (L), quadratic features (Q), hinge features (H), product features (P), and threshold features (T), this study constructed 527 candidate models. The optimal model was selected based on the delta values derived from the Akaike's Information criterion corrected (AICc). The model with the minimum AICc value (ΔAICc = 0) is considered the optimal one (Cobos *et al.*, 2019). In this study, the optimized parameters for the MaxEnt software were set as RM = 5 and FC = LQPTH.

**Supporting Method**

**1.Research Method**

**1.1 Experimental Materials and Instruments**

**1.1.1 Samples**

**Table M1. Information of the test samples of the Chinese herbal medicine *E. senticosus***

| Serial Number | Batch Number | Number of Samples | Sample Weight |
| --- | --- | --- | --- |
| 1 | ES-SZ-07 | AZ2024100076 | 480g |
| 2 | ES-TW-09 | AZ2024100077 | 465g |
| 3 | ES-JY-11 | AZ2024100078 | 485g |
| 4 | ES-MX-13 | AZ2024100079 | 490g |
| 5 | ES-NC-15 | AZ2024100080 | 465g |
| 6 | ES-WY-17 | AZ2024100081 | 565g |
| 7 | ES-YH-19 | AZ2024100082 | 480g |
| 8 | ES-BL-20 | AZ2024100083 | 470g |
| 9 | ES-LX-24 | AZ2024100084 | 405g |
| 10 | ES-WC-25 | AZ2024100085 | 445g |
| 11 | ES-WDLC-29 | AZ2024100091 | 470g |
| 12 | ES-HL-54 | AZ2024100092 | 515g |
| 13 | ES-FZ-35 | AZ2024100093 | 480g |
| 14 | ES-FZ-41 | AZ2024100094 | 510g |
| 15 | ES-FZ-47 | AZ2024100095 | 485g |
| 16 | ES-TY-48 | AZ2024100096 | 575g |
| 17 | ES-YL-50 | AZ2024100097 | 495g |
| 18 | ES-HG-52 | AZ2024100098 | 510g |
| 19 | ES-AC-31 | AZ2024100099 | 500g |
| 20 | ES-BQ-57 | AZ2024100100 | 480g |
| 21 | ES-MS-61 | AZ2024100101 | 470g |
| 22 | ES-LK-65 | AZ2024100102 | 450g |
| 23 | ES-ML-67 | AZ2024100103 | 500g |
| 24 | ES-SFH-68 | AZ2024100104 | 480g |
| 25 | ES-MDJ-72 | AZ2024100105 | 465g |
| 26 | ES-HL-73 | AZ2024100106 | 480g |
| 27 | ES-WC-83 | AZ2024100107 | 475g |
| 28 | ES-WC-87 | AZ2024100108 | 480g |
| 29 | ES-SZ-89 | AZ2024100109 | 445g |
| 30 | ES-SZ-91 | AZ2024100110 | 460g |
| 31 | ES-QA-96 | AZ2024100111 | 505g |
| 32 | ES-SL-98 | AZ2024100112 | 475g |
| 33 | ES-WMH-103-01 | AZ2024100114 | 500g |
| 34 | ES-TL-104-01 | AZ2024100115 | 500g |
| 35 | ES-LB-105-01 | AZ2024100116 | 500g |
| 36 | ES-HN-106-01 | AZ2024100117 | 500g |

**1.1.2 Reference Substance / Reference Herb**

**Table M2. Information on reference substances/reference herbs**

| Serial Number | Name of Medicinal Materials/Drugs | Manufacturers | Batch Number |
| --- | --- | --- | --- |
| 1 | *E. Senticosus* Reference Material | National Institutes for Food and Drug Control, China | 120991-202111 |
| 2 | Syringin | National Institutes for Food and Drug Control, China | 111574-202106 |
| 3 | Eleutheroside E | National Institutes for Food and Drug Control, China | 111713-202105 |
| 4 | Eleutheroside B1 | Naito Biotech (Shanghai) Co., Ltd. | 030158-202311 |
| 5 | Isofraxidin | National Institutes for Food and Drug Control, China | 110837-202310 |
| 6 | Protocatechuic Acid | National Institutes for Food and Drug Control, China | 110809-201906 |
| 7 | Coniferin | Naito Biotech (Shanghai) Co., Ltd. | 190233-202201 |
| 8 | Syringaresinol-4'-O-*β*-D-glucopyranoside | Shanghai Standard Technology Service Co., Ltd. | 19638-G241001 |

**1.1.3 Reagents and Test Drugs**

**Table M3. Information on reagents and test drugs**

| Serial Number | Drug Name | Manufacturers | Batch Number | Grade |
| --- | --- | --- | --- | --- |
| 1 | Methanol | Dima | LOT:R142743 | Chromatographic Grade |
| 2 | Acetonitrile | Dima | LOT:R142750 | Chromatographic Grade |
| 3 | Methanol | Chih Yuen | 20240701 | Analytical Grade |
| 4 | Phosphoric Acid | Chih Yuen | 20230801424 | Analytical Grade |
| 5 | Methanoic Acid | Chih Yuen | 20230501327 | Analytical Grade |
| 6 | Ultrapure Water | Self-prepared | —— | —— |

**1.1.4 Instruments and Equipment**

**Table M4. Information of instruments and equipment**

| Serial Number | Instrument Name | Version | Manufacturers | Instrument number |
| --- | --- | --- | --- | --- |
| 1 | HPLC(high performance liquid chromatograph) | E2695/PDA2998 | Waters | AG-01-102 |
| 2 | HPLC(high performance liquid chromatograph) | E2695/UV2489 | Waters | AG-01-107 |
| 3 | Chromatographic column | Agilent 5HC（2）-C18 | Agilent | AG-LC-151 |
| 4 | Chromatographic column | Waters Atlantis TM T3 | Waters | AG-Y-005 |
| 5 | Chromatographic column | Inertsil ODS-3 C18 | Shimadzu | AG-Y-002 |

**1.2 Establishment of Analytical Methods**

**1.2.1 Selection of Chromatographic Conditions**

Approximately 1 g of coarse powder of *E. senticosus* was accurately weighed and transferred into a stoppered conical flask. Then, 25 mL of 50% methanol was precisely added. The flask was tightly sealed and weighed. The mixture was ultrasonicated (power: 250 W; frequency: 50 kHz) for 30 minutes. After cooling to room temperature, the flask was reweighed, and the weight loss was compensated by adding the same extraction solvent. The mixture was shaken thoroughly and filtered. The subsequent filtrate was passed through a 0.45 μm organic filter membrane to obtain the final sample solution.

**Table M5. Comparison of initial chromatographic conditions**

| Scheme | Mobile Phase | Elution Program | Column Temperature ℃ | Flow Rate mL/min | Basis for Formulation |
| --- | --- | --- | --- | --- | --- |
| 1 | Acetonitrile (mobile phase A) - 0.1% Phosphoric Acid Solution (mobile phase B) | The gradient elution program is shown in Table M6. | 30 | 0.8 | Luo Xu;Tang Xiaolong;Yang Songqi;Chen Haiyan;Ke Xiao;Sichuan Jishengtang Pharmaceutical Co.Ltd.;. HPLC Fingerprint of *Acanthopanax Senticosus* from Different Habitats[J]. China Pharmaceuticals, 2017, 26(20): 6-8. |
| 2 | Acetonitrile (mobile phase A) - Water (mobile phase B) | The gradient elution program is shown in Table M7. | 35 | 1.0 | SONG Juan;ZHANG Jingyu;LYU Chongning;LU Jincai;School of Traditional Chinese Materia Medica,Shenyang Pharmaceutical University;. Quality evaluation of *Acanthopanax senticosus* based on HPLC fingerprint and multi-component content determination[J]. Chinese Traditional and Herbal Drugs, 2024, 55(04): 1326-1333. |
| 3 | Methanol (mobile phase A) - 0.1% Methanoic acid aqueous solution (mobile phase B) | The gradient elution program is shown in Table M8. | 25 | 0.8 | PAN Haifeng,LI Yan-rong.Study on the Roots and Stems Fingerprinting of *Acanthopanax senticosus* Harms by HPLC[J]. Journal of Chinese Pharmaceutical Sciences, 2010, 45(15): 1136-1139. |
| 4 | Acetonitrile (mobile phase A) - 0.2% Methanoic acid aqueous solution (mobile phase B) | The gradient elution program is shown in Table M6. | 30 | 0.8 | Luo Xu;Tang Xiaolong;Yang Songqi;Chen Haiyan;Ke Xiao;Sichuan Jishengtang Pharmaceutical Co.Ltd.;. HPLC Fingerprint of *Acanthopanax Senticosus* from Different Habitats[J]. China Pharmaceuticals, 2017, 26(20): 6-8. |
| 5 | Acetonitrile (mobile phase A) - 0.2% Methanoic acid aqueous solution (mobile phase B) | The gradient elution program is shown in Table M7. | 35 | 1.0 | SONG Juan;ZHANG Jingyu;LYU Chongning;LU Jincai;School of Traditional Chinese Materia Medica,Shenyang Pharmaceutical University;. Quality evaluation of *Acanthopanax senticosus* based on HPLC fingerprint and multi-component content determination[J]. Chinese Traditional and Herbal Drugs, 2024, 55(04): 1326-1333. |
| 6 | Acetonitrile (mobile phase A) - Water (mobile phase B) | The gradient elution program is shown in Table M6. | 30 | 0.8 | Luo Xu;Tang Xiaolong;Yang Songqi;Chen Haiyan;Ke Xiao;Sichuan Jishengtang Pharmaceutical Co.Ltd.;. HPLC Fingerprint of *Acanthopanax Senticosus* from Different Habitats[J]. China Pharmaceuticals, 2017, 26(20): 6-8. |

**Table M6. The gradient elution program of scheme 1,4,6**

| Time (min) | Mobile Phase A % | Mobile Phase B % |
| --- | --- | --- |
| 0 | 7 | 93 |
| 1 | 8 | 92 |
| 10 | 10 | 90 |
| 23 | 15 | 85 |
| 53 | 30 | 70 |
| 63 | 45 | 55 |
| 73 | 45 | 55 |

**Table M7. The gradient elution program of scheme 2,5**

| Time (min) | Mobile Phase A % | Mobile Phase B % |
| --- | --- | --- |
| 0 | 2 | 98 |
| 20 | 10 | 90 |
| 30 | 13 | 87 |
| 55 | 18 | 82 |
| 60 | 2 | 98 |

**Table M8. The gradient elution program of scheme 3**

| Time (min) | Mobile Phase A % | Mobile Phase B % |
| --- | --- | --- |
| 0 | 20 | 80 |
| 25 | 20 | 80 |
| 45 | 23 | 77 |
| 50 | 28 | 72 |
| 75 | 30 | 70 |
| 80 | 35 | 65 |
| 85 | 38 | 62 |
| 120 | 46 | 54 |
| 150 | 55 | 45 |

**1.2.1.1 Experiment Results**

One batch of *E. Senticosus* (origin information of the test sample is provided in Table M9) was selected. According to the sample pretreatment conditions described in schemes 1 to 6 under Section 1.2.1, test sample solutions were prepared. Subsequently, experiments were conducted under the respective chromatographic conditions specified in schemes 1 to 6. A photodiode array (PDA) detector was used for full-wavelength scanning, and chromatograms at specific wavelengths were recorded.

**Table M9. Information on test samples for chromatographic condition comparison**

| Serial Number | Sample Name | Number of Samples | Source of Samples |
| --- | --- | --- | --- |
| 1 | *E. Senticosus* | AZ2024100117 | Heilongjiang Province |

**Fig. M1. Chromatograms of preliminary experimental results for schemes 1 to 6**

(Note: S1-S9 correspond to Scheme 1 at 210 nm, Scheme 1 at 340 nm, Scheme 2 at 210 nm, Scheme 2 at 340 nm, Scheme 3 at 210 nm, Scheme 3 at 340 nm, Scheme 4, Scheme 5, and Scheme 6, respectively.)

**Fig. M2. Full-wavelength scanning chromatogram for scheme 1**

**1.2.1.2 Results Analysis**

Based on the evaluation of peak shape, baseline stability, separation efficiency, and number of detectable peaks, an appropriate chromatographic scheme was selected, Figs M2 and M3 indicate that under scheme 1 at 210 nm, the gradient elution time was appropriate, the baseline remained stable, chromatographic peaks contained abundant information, and overall resolution was satisfactory. Therefore, scheme 1 is considered the preferred elution condition for method development.

**1.2.1.3 Experiment Conclusion**

Scheme 1 was selected as the foundational chromatographic condition for subsequent experiments, with the detection wavelength set at 210 nm.

**1.2.2 Reference Substance Positioning and Optimization**

Appropriate amounts of protocatechuic acid, coniferin, syringin, eleutheroside B1, eleutheroside E, isofraxidin, and syringaresinol-4'-O-*β*-D-glucopyranoside reference standards were accurately weighed separately and transferred into volumetric flasks. Each standard was dissolved in methanol to prepare individual reference solutions with a concentration of 0.1 mg/mL. Five batches of test samples were selected and analyzed according to the chromatographic conditions described in Section 1.2.1 to verify the universality of the method. The results are presented as follows:

**Table M10. Information of test samples for comparison of chromatographic conditions**

| Serial Number | Batch Number | Number of Samples | Source of Samples |
| --- | --- | --- | --- |
| 1 | ES-YL-50 | AZ2024100097 | Heilongjiang Province |
| 2 | ES-HG-52 | AZ2024100098 | Heilongjiang Province |
| 3 | ES-BQ-57 | AZ2024100100 | Heilongjiang Province |
| 4 | ES-SL-98 | AZ2024100112 | Heilongjiang Province |
| 5 | ES-HN-106-01 | AZ2024100117 | Heilongjiang Province |

**1.2.2.1 Experimental Result：**

**Fig. M3. Chromatograms obtained from reference compound localization**

(Note: S1–S13 correspond to *E. Senticosus* batches AZ2024100097, AZ2024100098, AZ2024100100, AZ2024100112, AZ2024100117, mixed reference standards, protocatechuic acid, coniferin, syringin, eleutheroside B1, eleutheroside E, isofraxidin, and syringaresinol-4'-O-*β*-D-glucopyranoside, respectively.)

Corresponding chromatographic peaks were detected in the test solution at retention times matching those of the reference standards, namely protocatechuic acid, coniferin, syringin, eleutheroside B1, eleutheroside E, isofraxidin, and syringaresinol-4'-O-*β*-D-glucopyranoside. Methanol was used as the solvent for preparation, and this solvent system yielded symmetrical peak shapes free of solvent interference. Based on the comparison of the corresponding peak areas between the reference solution and the test solution, the composition of the reference solution was optimized to a methanol-prepared mixed standard solution containing 10 μg/mL each of protocatechuic acid, coniferin, eleutheroside B1, isofraxidin, and syringaresinol-4'-O-*β*-D-glucopyranoside, along with 40 μg/mL syringin and 20 μg/mL eleutheroside E.

**Fig. M4. Chromatogram obtained after optimization of reference standard concentrations.**

(Note: S1 and S2 correspond to the mixed reference standard and *E. Senticosus* sample AZ2024100117, respectively.)

**1.2.2.2 Results Analysis**

As shown in the figure, chromatographic peaks corresponding to protocatechuic acid, coniferin, syringin, eleutheroside B1, eleutheroside E, isofraxidin, and syringaresinol-4'-O-*β*-D-glucopyranoside were unambiguously identified at retention times matching those of their respective reference standards. The mixed reference standard solution, consisting of the aforesaid components, was prepared in methanol at the optimized concentrations.

**1.2.2.3 Experimental Conclusions**

Under the established chromatographic conditions, the method enabled the clear identification of the chromatographic peaks corresponding to protocatechuic acid, coniferin, syringin, eleutheroside B1, eleutheroside E, isofraxidin, and syringaresinol-4'-O-*β*-D-glucopyranoside. The mixed reference standard solution was prepared in methanol, with concentrations of 10 μg/mL for protocatechuic acid, coniferin, eleutheroside B1, isofraxidin, and syringaresinol-4'-O-*β*-D-glucopyranoside; 40 μg/mL for syringin; and 20 μg/mL for eleutheroside E.

**1.2.3 Optimization of the Preparation of Test Solution**

**1.2.3.1 Evaluation of Extraction Solvents (Factors investigated: 50% methanol, 80%**

**methanol, and pure methanol)**

**1.2.3.1.1 Sample Preparation:**

A single batch of *E. senticosus* (AZ2024100117) was used as the test material. The effects of different extraction solvents were compared based on evaluation indicators such as the number of chromatographic peaks and resolution. The solvents tested included 50% methanol, 80% methanol, and pure methanol.

Preparation of the Test Solution: Approximately 1 g of coarse powder of *E. Senticosus* was accurately weighed and transferred into a stoppered conical flask. Then, 25 mL of the corresponding extraction solvent (as specified in the methodology) was precisely added. The flask was stoppered, and the total weight was recorded. The mixture was subjected to ultrasonic extraction (power: 250 W, frequency: 50 kHz) for 30 minutes. After cooling to room temperature, the weight was recorded again, and the weight loss was compensated by adding the same solvent. The mixture was shaken well, filtered, and the subsequent filtrate was passed through a 0.45 μm organic filter membrane to obtain the final test solution.

**1.2.3.1.2 Experimental Results:**

**Fig. M5. Chromatograms obtained from the evaluation of extraction solvents**

(Note: S1, S2, and S3 represent the extracts obtained with 50% methanol, 80% methanol, and pure methanol, respectively.)

**Table M11. Comparison of peak areas and specific peak areas (per unit mass) obtained with different extraction solvents**

| Comparison of Peak Areas across Different Extraction Solvents | | | | Comparison of Peak Area Per Unit Mass with Different Extraction Solvents | | | |
| --- | --- | --- | --- | --- | --- | --- | --- |
| Peak Sequence Number | 50% methanol | 80% methanol | pure methanol | Peak Sequence Number | 50% methanol | 80% methanol | pure methanol |
| Peak 1 | 112548 | 161157 | 124915 | Peak 1 | 104086 | 155108 | 123336 |
| Peak 2 | 263500 | 295976 | 249153 | Peak 2 | 243688 | 284866 | 246004 |
| Peak 3 | 1361395 | 1530649 | 1325204 | Peak 3 | 1259035 | 1473194 | 1308456 |
| Peak 4 | 327534 | 390265 | 313785 | Peak 4 | 302908 | 375616 | 309819 |
| Peak 5 | 1587101 | 1561689 | 1321643 | Peak 5 | 1467771 | 1503069 | 1304940 |
| Peak 6 | 570265 | 516131 | 496901 | Peak 6 | 527388 | 496757 | 490621 |
| Peak 7 | 145533 | 136868 | 118678 | Peak 7 | 134591 | 131731 | 117178 |

**1.2.3.1.3 Result Analysis:**

Chromatographic analysis revealed that the resolution and theoretical plate numbers of the peaks were comparable when using 50% methanol, 80% methanol, or pure methanol as the extraction solvent; however, the extraction effect of 80% methanol was better when comparing the peak area per unit mass. Consequently, 80% methanol was selected as the optimal extraction solvent.

**1.2.3.1.4 Experimental Conclusion:**

80% methanol was selected as the extraction solvent.

**1.2.3.2 Optimization of Extraction Method and Duration (Factors investigated: ultrasonic extraction for 20, 30, and 50 minutes; reflux extraction for20, 30, and 50 minutes)**

**1.2.3.2.1 Sample Preparation:**

Based on the previously determined optimal extraction solvent, the effects of different extraction methods and durations were investigated to identify the most effective protocol. Ultrasonic extraction was performed for 20, 30, and 50 minutes, whereas reflux extraction was conducted for 20, 30, and 50 minutes. The specific peak areas (normalized per unit mass) of the major chromatographic peaks served as the primary evaluation criterion for selecting the appropriate method and duration.

Approximately 1 g of coarse powder of *E. senticosus* was accurately weighed and transferred into a stoppered conical flask. Then, 25 mL of 80% methanol was precisely added, the flask was sealed, and its total weight was recorded. Extraction was performed according to the predefined processing conditions (ultrasonic treatment for 20, 30, or 50 minutes; reflux extraction for 20, 30, or 50 minutes). After cooling to room temperature, the weight was measured again, and any loss was compensated by adding 80% methanol. The mixture was thoroughly shaken, filtered, and the subsequent filtrate was passed through a 0.45 μm organic filter membrane to obtain the final test solution.

**1.2.3.2.2 Experimental Results:**

**Fig. M6. Chromatograms obtained under different extraction durations**

(Note: S1 to S6 represent the extracts obtained by ultrasonic extraction for 20, 30, and 50 minutes, and reflux extraction for 20, 30, and 50 minutes, respectively.)

**Table M12. Comparison of peak areas and specific peak areas (per unit mass) under different extraction methods and durations**

| Comparison of Peak Areas under Different Extraction Methods and Durations | | | | | | |
| --- | --- | --- | --- | --- | --- | --- |
| Peak Sequence Number  numberequenceodTimePeak sequence number  Method and Times | Ultrasonic Extraction 20 min | Ultrasonic Extraction 30 min | Ultrasonic Extraction 50 min | Reflux Extraction 20 min | Reflux Extraction 30 min | Reflux Extraction 50 min |
| Peak 1 | 153411 | 157224 | 166946 | 228505 | 211433 | 209361 |
| Peak 2 | 295497 | 302576 | 302297 | 321921 | 306490 | 288277 |
| Peak 3 | 1557380 | 1582386 | 1576643 | 1650766 | 1551357 | 1452226 |
| Peak 4 | 399046 | 398136 | 386583 | 409280 | 386146 | 349697 |
| Peak 5 | 1492341 | 1586631 | 1654099 | 1851861 | 1823352 | 1840836 |
| Peak 6 | 493217 | 520033 | 539711 | 563792 | 545161 | 537368 |
| Peak 7 | 129239 | 136982 | 145828 | 170889 | 179038 | 180311 |
| Comparison of Specific Peak Areas (per Unit Mass) under Different Extraction Methods and Durations | | | | | | |
| Peak Sequence Number  Method and Times | Ultrasonic Extraction 20 min | Ultrasonic Extraction 30 min | Ultrasonic Extraction 50 min | Reflux Extraction 20 min | Reflux Extraction 30 min | Reflux Extraction 50 min |
| Peak 1 | 153028 | 154414 | 163914 | 223981 | 206902 | 202850 |
| Peak 2 | 294760 | 297168 | 296806 | 315547 | 299922 | 279311 |
| Peak 3 | 1553496 | 1554101 | 1548005 | 1618081 | 1518110 | 1407059 |
| Peak 4 | 398051 | 391019 | 379561 | 401176 | 377871 | 338821 |
| Peak 5 | 1488619 | 1558270 | 1624054 | 1815194 | 1784276 | 1783583 |
| Peak 6 | 491987 | 510738 | 529908 | 552629 | 533478 | 520655 |
| Peak 7 | 128917 | 134533 | 143179 | 167505 | 175201 | 174703 |

**1.2.3.2.3 Results Analysis:**

Although all six extraction protocols resulted in comparable chromatographic resolution and theoretical plate numbers, the reflux extraction for 20 minutes demonstrated superior performance based on a comparison of peak areas per unit mass. Therefore, reflux extraction for 20 minutes was designated as the optimal sample pretreatment method.

**1.2.3.2.4 Experimental Conclusion:**

Reflux extraction for 20 minutes was adopted as the method for sample pretreatment.

**1.2.3.3 Optimization of Sample Quantity**

**1.2.3.3.1 Investigation of Sample Mass (Tested factors: 0.5 g, 1.0 g, and 1.5 g)**

Based on the previously optimized extraction time, the effect of solid–liquid ratio was investigated to determine the optimal sample mass. Sample masses of 0.5 g, 1.0 g, and 1.5 g were evaluated. The number of chromatographic peaks, resolution, and the peak areas per unit mass of the major peaks were used as the key criteria for selecting the appropriate sample mass.

An appropriate amount of coarse powder of *E. senticosus* (according to the designated sample mass) was accurately weighed and transferred into a stoppered conical flask. Then, 25 mL of 80% methanol was precisely added, the flask was sealed tightly, and its total weight was recorded. The mixture was subjected to reflux extraction for 20 minutes. After cooling to room temperature, the weight was measured again, and any loss was compensated by adding 80% methanol. The mixture was shaken thoroughly, filtered, and the subsequent filtrate was passed through a 0.45 μm organic filter membrane to obtain the final test solution.

**1.2.3.3.2 Experimental Results:**

**Fig. M7. Chromatograms from the investigation of different sample masses**

(Note: S1 to S3 represent sample masses of 0.5 g, 1.0 g, and 1.5 g, respectively.)

**Table M13. Comparison of peak areas and specific peak areas (per unit mass) under different solid-to-liquid ratios**

| Comparison of Peak Areas using Different Extraction Solvents | | | | Comparison of Specific Peak Areas using Different Extraction Solvents | | | |
| --- | --- | --- | --- | --- | --- | --- | --- |
| Peak sequence number  Solid liquid  ratio | 0.5 g | 1.0 g | 1.5 g | Peak sequence number  Solid liquid  ratio | 0.5 g | 1.0 g | 1.5 g |
| Peak 1 | 100202 | 206784 | 315969 | Peak 1 | 196090 | 206083 | 209695 |
| Peak 2 | 147485 | 313309 | 441154 | Peak 2 | 288620 | 312247 | 292775 |
| Peak 3 | 759130 | 1586265 | 2261036 | Peak 3 | 1485577 | 1580890 | 1500555 |
| Peak 4 | 188051 | 400745 | 556137 | Peak 4 | 368006 | 399387 | 369085 |
| Peak 5 | 889169 | 1786584 | 2592346 | Peak 5 | 1740057 | 1780530 | 1720431 |
| Peak 6 | 259611 | 531981 | 777134 | Peak 6 | 508045 | 530178 | 515751 |
| Peak 7 | 83963 | 166020 | 260925 | Peak 7 | 164311 | 165457 | 173165 |

**1.2.3.3.3 Results Analysis:**

Based on the analytical data, a sample mass of 1.0 g yielded a more complete extraction and was therefore selected as the optimal condition.

**1.2.3.3.4 Experimental Conclusion:**

Preparation of Test Solution:An accurately weighed portion of approximately 1.0 g of *E. senticosus* coarse powder was transferred into a stoppered conical flask. Then, 25 mL of 80% methanol was precisely added, the flask was sealed tightly, and its total weight was recorded. The mixture was subjected to reflux extraction for 20 minutes. After cooling to room temperature, the weight was measured again, and the loss of solvent was replenished with 80% methanol. The solution was shaken thoroughly, filtered, and the subsequent filtrate was passed through a 0.45 μm organic filter membrane to obtain the test solution.

**1.2.4 Method Validation and System Suitability**

**1.2.4.1 Integrity Test**

Record the fingerprint chromatogram for a duration longer than twice the preset measurement time to examine whether the main components contained in the sample are reflected in the chromatogram and whether the criterion of maximizing effective information is satisfied. The experimental results are presented in the figure below, demonstrating that complete peak information was fully acquired within 160 minutes.

**Fig. M8. Chromatogram of the test solution used to integrity test.**

**1.2.4.2 Confirmation of Major Chromatographic Peaks**

**1.2.4.2.1: Take the reference substance solutions prepared in "1.2.2" for injection, and record the identification of the main chromatographic peaks.**

**1.2.4.2.2: Experimental Results:**

**Fig. M9. Identification of major chromatographic peaks – chromatograms of reference and test samples**

(Note: S1 to S9 correspond to protocatechuic acid, coniferin, syringin, eleutheroside B1, eleutheroside E, isofraxidin, syringaresinol-4'-O-*β*-D-glucopyranoside, mixed reference standards, and *E. senticosus* sample AZ2024100117, respectively.)

**1.2.4.2.3: Result Analysis**

The components of protocatechuic acid, coniferin, syringin, eleutheroside B1, eleutheroside E, isofraxidin, and syringaresinol-4'-O-*β*-D-glucopyranoside can be identified by the reference standard retention time method.

**1.2.4.3 System Suitability Test**

**1.2.4.3.1 Determination Method**

The analysis was performed via high-performance liquid chromatography (HPLC) according to Chinese Pharmacopoeia General Chapter 0512.

Chromatographic Conditions and System Suitability Test: The stationary phase was octadecylsilica gel. The mobile phase consisted of acetonitrile (A) and 0.1% phosphoric acid (B) solution, and gradient elution was performed according to the gradient program specified in the table below. The detection wavelength was 210 nm. The column temperature was 30 ℃. The flow rate was 0.8 mL/min.

**Table M14. Gradient elution program of the mobile phase**

| Time (min) | Mobile Phase A% | Mobile Phase B% |
| --- | --- | --- |
| 0 | 7 | 93 |
| 1 | 8 | 92 |
| 10 | 10 | 90 |
| 23 | 15 | 85 |
| 53 | 30 | 70 |
| 63 | 45 | 55 |
| 73 | 45 | 55 |
| 73.1 | 7 | 93 |
| 80 | 7 | 93 |

Preparation of the Reference Solution: Approximately 1.0 g of the *E. senticosus* reference herbal material was accurately weighed and transferred into a stoppered conical flask. Then, 25 mL of 80% methanol was precisely added. The flask was stoppered and weighed. The mixture was subjected to heating under reflux for 20 minutes, then allowed to cool to room temperature, and weighed again. The weight loss was compensated by adding 80% methanol. After shaking thoroughly, the solution was filtered. The subsequent filtrate was passed through a 0.45 μm organic filter membrane to obtain the final reference herbal material solution. Separately, appropriate amounts of protocatechuic acid, coniferin, syringin, eleutheroside B1, eleutheroside E, isofraxidin, and syringaresinol-4'-O-*β*-D-glucopyranoside were accurately weighed and dissolved in methanol to prepare a mixed reference standard solution. The final solution contained protocatechuic acid, coniferin, eleutheroside B1, isofraxidin, and syringaresinol-4'-O-*β*-D-glucopyranoside at concentrations of 10 μg/mL each, syringin at 40 μg/mL, and eleutheroside E at 20 μg/mL.

Preparation of the Test Solution: Approximately 1.0 g of coarse powder of *E. senticosus* was accurately weighed and transferred into a stoppered conical flask. Then, 25 mL of 80% methanol was added precisely. The flask was stoppered, weighed, and then heated under reflux for 20 minutes. After cooling to room temperature, the flask was weighed again, and the loss in weight was compensated by adding 80% methanol. The mixture was shaken well and filtered. The resulting filtrate was passed through a 0.45 μm organic filter membrane to obtain the final test solution.

Determination: Aliquots (10 μL each) of both the reference solution and the test solution were separately injected into the liquid chromatograph for analysis.

Following the described method, the injections demonstrated symmetrical peaks and good resolution for both the reference standards and the test sample. The chromatogram of the test sample showed peaks that were consistent with the retention times of the respective reference standards.

Analysis was performed according to the method described in section 1.2.4.3.1. The chromatograms showed symmetrical peaks and satisfactory resolution for both the reference standards and the test sample. Peaks were detected in the test solution at retention times consistent with those of protocatechuic acid, coniferin, syringin, eleutheroside B1, eleutheroside E, isofraxidin, and syringaresinol-4'-O-*β*-D-glucopyranoside. The number of theoretical plates, calculated based on the eleutheroside E peak, was not less than 2000.

**1.2.5 Establishment of Reference Fingerprint Profiles**

**1.2.5.1 Establishment of reference chromatograms and identification of common peaks**

According to the requirements of the National Drug Standard Working Manual (Fourth Edition), the samples used for establishing the reference fingerprint chromatogram should be representative. Therefore, fifteen batches of *E. senticosus* crude drug (for detailed sample information, see Table M15) were selected and analyzed following the chromatographic conditions and sample processing procedure described in section 1.2.4.3.1.

**Table M15. Sample information**

| Serial Number | Number of Samples | Sample Name | Batch Number | Source of Samples |
| --- | --- | --- | --- | --- |
| 1 | AZ2024100078 | *E. senticosus* | ES-JY-11 | Heilongjiang Province |
| 2 | AZ2024100082 | *E. senticosus* | ES-YH-19 | Heilongjiang Province |
| 3 | AZ2024100094 | *E. senticosus* | ES-FZ-41 | Heilongjiang Province |
| 4 | AZ2024100097 | *E. senticosus* | ES-YL-50 | Heilongjiang Province |
| 5 | AZ2024100098 | *E. senticosus* | ES-HG-52 | Heilongjiang Province |
| 6 | AZ2024100099 | *E. senticosus* | ES-AC-31 | Heilongjiang Province |
| 7 | AZ2024100100 | *E. senticosus* | ES-BQ-57 | Heilongjiang Province |
| 8 | AZ2024100102 | *E. senticosus* | ES-LK-65 | Heilongjiang Province |
| 9 | AZ2024100107 | *E. senticosus* | ES-WC-83 | Heilongjiang Province |
| 10 | AZ2024100110 | *E. senticosus* | ES-SZ-91 | Heilongjiang Province |
| 11 | AZ2024100112 | *E. senticosus* | ES-SL-98 | Heilongjiang Province |
| 12 | AZ2024100114 | *E. senticosus* | ES-WMH-103-01 | Heilongjiang Province |
| 13 | AZ2024100115 | *E. senticosus* | ES-TL-104-01 | Heilongjiang Province |
| 14 | AZ2024100116 | *E. senticosus* | ES-LB-105-01 | Heilongjiang Province |
| 15 | AZ2024100117 | *E. senticosus* | ES-HN-106-01 | Heilongjiang Province |

**Fig. M10. Full-spectrum peak matching fitting results of 15 batches of test samples' chromatograms**

(S1–S15 correspond to the chromatograms of the test samples listed in Table M15; R represents the reference characteristic chromatogram generated by automated systemic alignment of the above 15 batches.)

The analysis was performed in accordance with the sample preparation procedure and chromatographic conditions. Using the "Traditional Chinese Medicine Chromatographic Fingerprint Similarity Evaluation System (2012 Edition)" software, a time window of 0.5 min was applied. The reference chromatogram was generated by the average method, and the full-spectrum peak matching was conducted.Following spectral alignment, a total of 39 common peaks were identified. Based on the chromatographic profiles of each sample, in addition to the seven components previously calibrated using reference standards (protocatechuic acid, coniferin, syringin, eleutheroside B1, eleutheroside E, isofraxidin, and syringaresinol-4'-O-*β*-D-glucopyranoside), four chromatographic peaks with good resolution, high peak areas and stable elution profiles were selected as characteristic peaks. These characteristic peaks were identified by comparing their relative retention times against the eleutheroside E reference peak. The chromatographic peaks from 15 batches of E. senticosus test samples showed corresponding retention times with those in the reference chromatogram of the control crude drug. Among these, eleven peaks specifically matched the retention times of their respective reference standards. The retention times of each characteristic peak and their relative retention times compared to the eleutheroside E peak (denoted as the S-peak) are presented in the table below.

**Table M16. Retention times of characteristic peaks in 15 batches of test samples**

| Batch Number/Retention Time | Peak 1 | Peak 2 | Peak 3 | Peak 4 | Peak 5 | Peak 6 | Peak 7(S) | Peak 8 | Peak 9 | Peak 10 | Peak 11 |
| --- | --- | --- | --- | --- | --- | --- | --- | --- | --- | --- | --- |
| Reference Herbs | 10.740 | 12.207 | 15.733 | 18.273 | 19.821 | 22.163 | 32.885 | 37.059 | 42.263 | 54.674 | 55.906 |
| ES-JY-11 | 10.809 | 12.261 | 15.825 | 18.367 | 19.890 | 22.238 | 32.901 | 37.000 | 42.253 | 54.645 | 55.873 |
| ES-YH-19 | 10.760 | 12.223 | 15.768 | 18.324 | 19.854 | 22.194 | 32.881 | 37.034 | 42.257 | 54.613 | 55.848 |
| ES-FZ-41 | 10.750 | 12.223 | 15.764 | 18.331 | 19.871 | 22.189 | 32.893 | 37.007 | 42.268 | 54.670 | 55.890 |
| ES-YL-50 | 10.777 | 12.240 | 15.769 | 18.343 | 19.875 | 22.217 | 32.878 | 37.061 | 42.237 | 54.629 | 55.857 |
| ES-HG-52 | 10.766 | 12.218 | 15.786 | 18.342 | 19.877 | 22.208 | 32.890 | 37.053 | 42.251 | 54.645 | 55.867 |
| ES-AC-31 | 10.783 | 12.240 | 15.777 | 18.319 | 19.867 | 22.194 | 32.883 | 37.043 | 42.234 | 54.674 | 55.915 |
| ES-BQ-57 | 10.846 | 12.292 | 15.878 | 18.432 | 19.956 | 22.294 | 32.934 | 37.113 | 42.288 | 54.695 | 55.931 |
| ES-LK-65 | 10.789 | 12.248 | 15.781 | 18.338 | 19.888 | 22.208 | 32.902 | 37.075 | 42.255 | 54.650 | 55.895 |
| ES-WC-83 | 10.792 | 12.257 | 15.798 | 18.343 | 19.879 | 22.185 | 32.849 | 37.061 | 42.209 | 54.609 | 55.839 |
| ES-SZ-91 | 10.754 | 12.234 | 15.751 | 18.286 | 19.838 | 22.135 | 32.836 | 37.013 | 42.170 | 54.545 | 55.768 |
| ES-SL-98 | 10.739 | 12.229 | 15.751 | 18.285 | 19.834 | 22.138 | 32.863 | 36.983 | 42.233 | 54.613 | 55.832 |
| ES-WMH-103-01 | 10.772 | 12.246 | 15.798 | 18.353 | 19.897 | 22.215 | 32.896 | 37.022 | 42.301 | 54.701 | 55.916 |
| ES-TL-104-01 | 10.821 | 12.275 | 15.842 | 18.398 | 19.945 | 22.268 | 32.893 | 37.056 | 42.242 | 54.633 | 55.858 |
| ES-LB-105-01 | 10.749 | 12.224 | 15.753 | 18.303 | 19.848 | 22.179 | 32.868 | 36.998 | 42.237 | 54.620 | 55.846 |
| ES-HN-106-01 | 10.756 | 12.222 | 15.748 | 18.297 | 19.840 | 22.180 | 32.851 | 36.976 | 42.220 | 54.622 | 55.840 |
| Retention Time RSD/% | 0.28 | 0.18 | 0.24 | 0.22 | 0.18 | 0.19 | 0.076 | 0.11 | 0.075 | 0.072 | 0.074 |

**Table M17. Relative retention times of characteristic peaks to the S-peak for 15 batches of test samples.**

| Batch Number/Relative Retention Time | Peak 1 | Peak 2 | Peak 3 | Peak 4 | Peak 5 | Peak 6 | Peak 7(S) | Peak 8 | Peak 9 | Peak 10 | Peak 11 |
| --- | --- | --- | --- | --- | --- | --- | --- | --- | --- | --- | --- |
| Reference Herbs | 0.327 | 0.371 | 0.478 | 0.556 | 0.603 | 0.674 | 1.000 | 1.127 | 1.285 | 1.663 | 1.700 |
| ES-JY-11 | 0.329 | 0.373 | 0.481 | 0.558 | 0.605 | 0.676 | 1.000 | 1.125 | 1.284 | 1.661 | 1.698 |
| ES-YH-19 | 0.327 | 0.372 | 0.480 | 0.557 | 0.604 | 0.675 | 1.000 | 1.126 | 1.285 | 1.661 | 1.698 |
| ES-FZ-41 | 0.327 | 0.372 | 0.479 | 0.557 | 0.604 | 0.675 | 1.000 | 1.125 | 1.285 | 1.662 | 1.699 |
| ES-YL-50 | 0.328 | 0.372 | 0.480 | 0.558 | 0.605 | 0.676 | 1.000 | 1.127 | 1.285 | 1.662 | 1.699 |
| ES-HG-52 | 0.327 | 0.371 | 0.480 | 0.558 | 0.604 | 0.675 | 1.000 | 1.127 | 1.285 | 1.661 | 1.699 |
| ES-AC-31 | 0.328 | 0.372 | 0.480 | 0.557 | 0.604 | 0.675 | 1.000 | 1.127 | 1.284 | 1.663 | 1.700 |
| ES-BQ-57 | 0.329 | 0.373 | 0.482 | 0.560 | 0.606 | 0.677 | 1.000 | 1.127 | 1.284 | 1.661 | 1.698 |
| ES-LK-65 | 0.328 | 0.372 | 0.480 | 0.557 | 0.604 | 0.675 | 1.000 | 1.127 | 1.284 | 1.661 | 1.699 |
| ES-WC-83 | 0.329 | 0.373 | 0.481 | 0.558 | 0.605 | 0.675 | 1.000 | 1.128 | 1.285 | 1.662 | 1.700 |
| ES-SZ-91 | 0.328 | 0.373 | 0.480 | 0.557 | 0.604 | 0.674 | 1.000 | 1.127 | 1.284 | 1.661 | 1.698 |
| ES-SL-98 | 0.327 | 0.372 | 0.479 | 0.556 | 0.604 | 0.674 | 1.000 | 1.125 | 1.285 | 1.662 | 1.699 |
| ES-WMH-103-01 | 0.327 | 0.372 | 0.480 | 0.558 | 0.605 | 0.675 | 1.000 | 1.125 | 1.286 | 1.663 | 1.700 |
| ES-TL-104-01 | 0.329 | 0.373 | 0.482 | 0.559 | 0.606 | 0.677 | 1.000 | 1.127 | 1.284 | 1.661 | 1.698 |
| ES-LB-105-01 | 0.327 | 0.372 | 0.479 | 0.557 | 0.604 | 0.675 | 1.000 | 1.126 | 1.285 | 1.662 | 1.699 |
| ES-HN-106-01 | 0.327 | 0.372 | 0.479 | 0.557 | 0.604 | 0.675 | 1.000 | 1.126 | 1.285 | 1.663 | 1.700 |
| Relative Retention Time RSD/% | 0.27 | 0.16 | 0.21 | 0.18 | 0.13 | 0.14 | 0 | 0.087 | 0.049 | 0.049 | 0.048 |

**1.2.5.2 Fitting with the Reference Spectrum**

**Fig. M11. The spectrum before fitting compared with the reference characteristic spectrum**

(S1-S15 represent the test sample batches as described above; R denotes the reference characteristic chromatogram automatically generated by the system through alignment of the aforementioned 15 batches.)

**Fig. M12. Aligned reference characteristic chromatogram**

(Peak 2: protocatechuic acid; Peak 3: coniferin; Peak 4: syringin; Peak 6: eleutheroside B1; Peak 7 (S): eleutheroside E; Peak 8: isofraxidin; Peak 9: syringaresinol-4'-O-*β*-D-glucopyranoside); Chromatographic column: Agilent 5HC-C18 (4.6 mm × 250 mm, 5 μm)

**1.2.6 Summary**

Through the aforementioned research findings, it was established that under chromatographic conditions based on literature methodology, a gradient elution procedure was employed using acetonitrile–0.1% phosphoric acid solution as the mobile phase. A systematic investigation of sample pretreatment methods was conducted, evaluating key parameters including extraction solvents, extraction techniques, extraction duration, and solid-to-liquid ratio. Following optimization, the sample preparation procedure delineated in Section 1.2.3.3.4 was ultimately adopted. For chromatographic separation, an Agilent 5HC-C18 (2) column (4.6 × 250 mm, 5 μm) with superior separation efficiency was selected for the experimental analysis.

A Summary of the developed methodology is presented below:

The analysis was performed via High-Performance Liquid Chromatography (HPLC) according to the Chinese Pharmacopoeia General Chapter 0512.

Chromatographic Conditions and System Suitability Test: An octadecylsilane bonded silica column was employed as the stationary phase. The mobile phase consisted of acetonitrile (A) and 0.1% phosphoric acid solution (B), with gradient elution performed according to the gradient program specified in the table. Detection was carried out at a wavelength of 210 nm. The column temperature was maintained at 30 °C, and the flow rate was set at 0.8  mL/min. The theoretical plate count, calculated based on the eleutheroside E peak, was required to be no less than 2000.

**Table M18. Mobile phase gradient elution program**

| Time (min) | Mobile Phase A% | Mobile Phase B% |
| --- | --- | --- |
| 0 | 7 | 93 |
| 1 | 8 | 92 |
| 10 | 10 | 90 |
| 23 | 15 | 85 |
| 53 | 30 | 70 |
| 63 | 45 | 55 |
| 73 | 45 | 55 |
| 73.1 | 7 | 93 |
| 80 | 7 | 93 |

Preparation of Reference Solution: Approximately 1.0 g of *E. senticosus* reference material was accurately weighed and transferred into a stoppered conical flask. Then, 25 mL of 80% methanol was precisely added, the flask was tightly sealed, and the total weight was recorded. The mixture was heated under reflux for 20 minutes, allowed to cool to room temperature, and weighed again. Any weight loss was compensated by adding 80% methanol. After thorough mixing, the solution was filtered, and the subsequent filtrate was passed through a 0.45 μm organic filter membrane to obtain the reference solution for the control medicinal material. A mixed reference solution was prepared by accurately weighing appropriate amounts of protocatechuic acid, coniferin, syringin, eleutheroside B1, eleutheroside E, isofraxidin, and syringaresinol-4'-O-*β*-D-glucopyranoside, followed by dissolution in methanol. The resulting solution contained protocatechuic acid, coniferin, eleutheroside B1, isofraxidin, and syringaresinol-4'-O-*β*-D-glucopyranoside at concentrations of 10 μg/mL each, syringin at 40 μg/mL, and eleutheroside E at 20 μg/mL.

Preparation of the Test Solution: Approximately 1.0 g of *E. senticosus* coarse powder was accurately weighed and transferred into a stoppered conical flask. Then, 25 mL of 80% methanol was precisely added, the flask was tightly sealed, and the total weight was recorded. The mixture was heated under reflux for 20 minutes, allowed to cool to room temperature, and weighed again. Any weight loss was compensated for by adding 80% methanol. After thorough mixing, the solution was filtered, and the subsequent filtrate was passed through a 0.45 μm organic filter membrane to obtain the test solution.

**1.3 Methodological Review**

The experimental procedures were conducted in accordance with the guidelines stipulated in the "Technical Requirements for the Study of Fingerprint Chromatograms of Traditional Chinese Medicine Injections (Provisional)" (hereinafter referred to as the "Technical Requirements"), as well as the "Explanation and Examples for the Establishment and Technical Requirements of Fingerprint Chromatograms of Chinese Medicinal Materials", "National Drug Standards Working Manual", "Chinese Pharmacopoeia", and other relevant regulatory documents. According to the technical requirements, during the establishment of the fingerprint chromatogram detection method, it is essential to select an appropriate detection method based on the characteristics of the test sample and the physicochemical properties of its chemical constituents. The rationale for the selection and the underlying principles of the method must be clearly explained. Additionally, the methodological validation data and relevant chromatograms, including parameters such as stability, precision, and reproducibility, should be established.

Stability Test: The stability of the test solution was evaluated by analyzing the identical sample at different time points. The consistency of the relative retention times and relative peak area ratios of the chromatographic peaks was assessed to determine the optimal detection time.

Precision Test: The precision of the instrument was evaluated by performing consecutive injections of the same test solution for at least five times, and the consistency of the relative retention times and relative peak area ratios of the chromatographic peaks was assessed. For the establishment of fingerprint chromatograms using high-performance liquid chromatography (HPLC) or gas chromatography (GC), the relative standard deviation (RSD) of the peak area ratios for the common peaks specified in the fingerprint chromatogram should not exceed 3%. For other analytical methods, the RSD of the peak area ratios should not exceed 5%.

Reproducibility Test: The reproducibility of the experimental method was evaluated by preparing and analyzing more than five test solutions from the same batch according to the established sample preparation and detection procedures. The consistency of the relative retention times and relative peak area ratios of the chromatographic peaks was assessed to validate the method's reproducibility. For the establishment of fingerprint chromatograms using high-performance liquid chromatography (HPLC) or gas chromatography (GC), the relative standard deviation (RSD) of the peak area ratios of the common characteristic peaks specified in the fingerprint should not exceed 3%. For other analytical methods, the corresponding RSD of the peak area ratios should not exceed 5%.

To evaluate the reliability of the analytical method, the test samples listed in the table below were used to conduct methodological validation for the following three parameters. Since the established method in this research is a characteristic chromatogram method, and the results are determined solely based on the relative retention times of each characteristic peak to the reference peak (S), the methodological validation only presents the variations in relative retention times, with no specific requirements for peak area ratios.

**Table M19. Information of test samples employed for methodological validation**

| Serial Number | Batch Number | Number of Samples | Source of Samples | Investigation Project |
| --- | --- | --- | --- | --- |
| 1 | ES-HN-106-01 | AZ2024100117 | Heilongjiang Province | Stability, precision, reproducibility, and robustness |

**1.3.1 Precision Test**

The precision of the analytical instrument was evaluated by performing six consecutive injections of the same test sample (AZ2024100117). The consistency of the relative retention times of the chromatographic peaks was assessed. The retention times of each characteristic peak and their relative retention times compared to the reference peak (S) are summarized in the table below. The relative standard deviation (RSD) of the relative retention times was found to be less than 2%.

**Table M20. Precision Test Data**

| Sample Items  Time | | Peak 1 | Peak 2 | Peak 3 | Peak 4 | Peak 5 | Peak 6 | Peak 7(S) | Peak 8 | Peak 9 | Peak 10 | Peak 11 |
| --- | --- | --- | --- | --- | --- | --- | --- | --- | --- | --- | --- | --- |
| Precision 1 | Retention Time | 10.724 | 12.190 | 15.705 | 18.254 | 19.802 | 22.141 | 32.798 | 36.927 | 42.196 | 54.639 | 55.847 |
|  | Relative Retention Time | 0.33 | 0.37 | 0.48 | 0.56 | 0.60 | 0.68 | 1.00 | 1.13 | 1.29 | 1.67 | 1.70 |
| Precision 2 | Retention Time | 10.727 | 12.195 | 15.696 | 18.242 | 19.792 | 22.126 | 32.792 | 36.920 | 42.182 | 54.605 | 55.821 |
|  | Relative Retention Time | 0.33 | 0.37 | 0.48 | 0.56 | 0.60 | 0.67 | 1.00 | 1.13 | 1.29 | 1.67 | 1.70 |
| Precision 3 | Retention Time | 10.751 | 12.215 | 15.732 | 18.284 | 19.833 | 22.166 | 32.801 | 36.932 | 42.186 | 54.614 | 55.830 |
|  | Relative Retention Time | 0.33 | 0.37 | 0.48 | 0.56 | 0.60 | 0.68 | 1.00 | 1.13 | 1.29 | 1.67 | 1.70 |
| Precision 4 | Retention Time | 10.775 | 12.233 | 15.766 | 18.316 | 19.863 | 22.200 | 32.842 | 36.957 | 42.204 | 54.609 | 55.818 |
|  | Relative Retention Time | 0.33 | 0.37 | 0.48 | 0.56 | 0.60 | 0.68 | 1.00 | 1.13 | 1.29 | 1.66 | 1.70 |
| Precision 5 | Retention Time | 10.766 | 12.233 | 15.757 | 18.303 | 19.856 | 22.181 | 32.816 | 36.947 | 42.190 | 54.599 | 55.826 |
|  | Relative Retention Time | 0.33 | 0.37 | 0.48 | 0.56 | 0.61 | 0.68 | 1.00 | 1.13 | 1.29 | 1.66 | 1.70 |
| Precision 6 | Retention Time | 10.773 | 12.226 | 15.763 | 18.319 | 19.858 | 22.191 | 32.790 | 36.915 | 42.178 | 54.612 | 55.823 |
|  | Relative Retention Time | 0.33 | 0.37 | 0.48 | 0.56 | 0.61 | 0.68 | 1.00 | 1.13 | 1.29 | 1.67 | 1.70 |
| Relative Retention Time RSD/% | | 0 | 0 | 0 | 0 | 0.86 | 0.61 | 0 | 0 | 0 | 0.31 | 0 |

**1.3.2 Reproducibility Test**

The reproducibility of the experimental methodology was evaluated using six independently prepared test samples from the same batch (AZ2024100117). Each sample was analyzed under the chromatographic conditions and sample preparation procedure described in Section 3.3.6. The consistency of the relative retention times of the chromatographic peaks was assessed. The retention times of each characteristic peak and their corresponding relative retention times compared to the reference peak (S) are presented in the table below. The relative standard deviation (RSD) of the relative retention times was determined to be less than 2%.

**Table M21 Data of Reproducibility Test**

| Sample Items  Time | | Peak 1 | Peak 2 | Peak 3 | Peak 4 | Peak 5 | Peak 6 | Peak 7(S) | Peak 8 | Peak 9 | Peak 10 | Peak 11 |
| --- | --- | --- | --- | --- | --- | --- | --- | --- | --- | --- | --- | --- |
| Reproducibility 1 | Retention Time | 10.843 | 12.279 | 15.877 | 18.432 | 19.964 | 22.293 | 32.842 | 36.951 | 42.199 | 54.655 | 55.868 |
|  | Relative Retention Time | 0.33 | 0.37 | 0.48 | 0.56 | 0.61 | 0.68 | 1.00 | 1.13 | 1.28 | 1.66 | 1.70 |
| Reproducibility 2 | Retention Time | 10.821 | 12.261 | 15.843 | 18.396 | 19.928 | 22.251 | 32.806 | 36.923 | 42.172 | 54.628 | 55.843 |
|  | Relative Retention Time | 0.33 | 0.37 | 0.48 | 0.56 | 0.61 | 0.68 | 1.00 | 1.13 | 1.29 | 1.67 | 1.70 |
| Reproducibility 3 | Retention Time | 10.793 | 12.241 | 15.809 | 18.366 | 19.900 | 22.226 | 32.813 | 36.923 | 42.187 | 54.654 | 55.863 |
|  | Relative Retention Time | 0.33 | 0.37 | 0.48 | 0.56 | 0.61 | 0.68 | 1.00 | 1.13 | 1.29 | 1.67 | 1.70 |
| Reproducibility 4 | Retention Time | 10.876 | 12.300 | 15.920 | 18.485 | 20.007 | 22.346 | 32.896 | 37.004 | 42.256 | 54.690 | 55.902 |
|  | Relative Retention Time | 0.33 | 0.37 | 0.48 | 0.56 | 0.61 | 0.68 | 1.00 | 1.12 | 1.28 | 1.66 | 1.70 |
| Reproducibility 5 | Retention Time | 10.846 | 12.282 | 15.883 | 18.432 | 19.957 | 22.284 | 32.840 | 36.965 | 42.218 | 54.670 | 55.878 |
|  | Relative Retention Time | 0.33 | 0.37 | 0.48 | 0.56 | 0.61 | 0.68 | 1.00 | 1.13 | 1.29 | 1.66 | 1.70 |
| Reproducibility 6 | Retention Time | 10.882 | 12.309 | 15.934 | 18.489 | 20.015 | 22.350 | 32.914 | 37.021 | 42.273 | 54.720 | 55.932 |
|  | Relative Retention Time | 0.33 | 0.37 | 0.48 | 0.56 | 0.61 | 0.68 | 1.00 | 1.12 | 1.28 | 1.66 | 1.70 |
| Relative Retention Time RSD/% | | 0 | 0 | 0 | 0 | 0 | 0 | 0 | 0.46 | 0.43 | 0.32 | 0 |

**1.3.3 Stability Test**

The stability of the test sample solution was evaluated by analyzing the same test sample (AZ2024100117) at designated time intervals (0 h, 6 h, 12 h, 18 h, and 24 h), and the consistency of the relative retention times of the chromatographic peaks was assessed. The retention times of the characteristic peaks and their corresponding relative retention times compared to the reference peak (S) are presented in the table below. The relative standard deviations (RSD) of the relative retention times were all less than 2%, confirming the stability of the sample solution within a 24-hour period.

**Table M22. Stability test data**

| Sample Items  Time | | Peak 1 | Peak 2 | Peak 3 | Peak 4 | Peak 5 | Peak 6 | Peak 7(S) | Peak 8 | Peak 9 | Peak 10 | Peak 11 |
| --- | --- | --- | --- | --- | --- | --- | --- | --- | --- | --- | --- | --- |
| 0 h | Retention Time | 10.794 | 12.260 | 15.803 | 18.348 | 19.886 | 22.224 | 32.814 | 36.929 | 42.181 | 54.652 | 55.867 |
|  | Relative Retention Time | 0.33 | 0.37 | 0.48 | 0.56 | 0.61 | 0.68 | 1.00 | 1.13 | 1.29 | 1.67 | 1.70 |
| 6 h | Retention Time | 10.858 | 12.310 | 15.906 | 18.458 | 19.992 | 22.321 | 32.865 | 36.979 | 42.206 | 54.676 | 55.891 |
|  | Relative Retention Time | 0.33 | 0.37 | 0.48 | 0.56 | 0.61 | 0.68 | 1.00 | 1.13 | 1.28 | 1.66 | 1.70 |
| 12 h | Retention Time | 10.850 | 12.300 | 15.886 | 18.441 | 19.977 | 22.295 | 32.836 | 36.960 | 42.192 | 54.654 | 55.864 |
|  | Relative Retention Time | 0.33 | 0.37 | 0.48 | 0.56 | 0.61 | 0.68 | 1.00 | 1.13 | 1.28 | 1.66 | 1.70 |
| 18 h | Retention Time | 10.832 | 12.289 | 15.880 | 18.438 | 19.969 | 22.306 | 32.883 | 36.986 | 42.219 | 54.684 | 55.901 |
|  | Relative Retention Time | 0.33 | 0.37 | 0.48 | 0.56 | 0.61 | 0.68 | 1.00 | 1.12 | 1.28 | 1.66 | 1.70 |
| 24 h | Retention Time | 10.841 | 12.291 | 15.866 | 18.415 | 19.958 | 22.285 | 32.840 | 36.959 | 42.190 | 54.652 | 55.859 |
|  | Relative Retention Time | 0.33 | 0.37 | 0.48 | 0.56 | 0.61 | 0.68 | 1.00 | 1.13 | 1.28 | 1.66 | 1.70 |
| Relative Retention Time RSD/% | | 0 | 0 | 0 | 0 | 0 | 0 | 0 | 0.40 | 0.35 | 0.27 | 0 |

**1.3.4 RobustnessTest**

The changes in the relative retention time and relative peak area of each characteristic peak were investigated when the detection wavelength varied within a small range (±2 nm), the column temperature changed within a small range (±2 °C), the flow rate changed within a small range (±0.1 mL/min), and different types of chromatographic columns were used. Given the complexity of the sample components and the frequent gradient changes in the method, variations in temperature, flow rate, and wavelength may lead to differences in separation efficiency. Therefore, these parameters should be carefully controlled. The impact of using different chromatographic columns on the relative retention times and relative peak area of the characteristic peaks was also assessed. The results indicated that different chromatographic columns affected both the response and separation efficiency of components in the test sample. Therefore, it is recommended to consistently employ the Agilent 5HC-C18 (2) column (4.6 × 250 mm, 5 μm) for this project. Considering the specific characteristics of the test sample and chromatographic conditions in this study, it is advised to use relative retention times as the criterion for evaluating the characteristic chromatogram, without imposing requirements for relative peak area ratios.

**Table M23. Robustness test data (different wavelengths)**

| Wavelength (nm)  Time | | Peak 1 | Peak 2 | Peak 3 | Peak 4 | Peak 5 | Peak 6 | Peak 7(S) | Peak 8 | Peak 9 | Peak 10 | Peak 11 |
| --- | --- | --- | --- | --- | --- | --- | --- | --- | --- | --- | --- | --- |
| 208 | Retention Time | 10.869 | 12.278 | 15.902 | 18.451 | 19.990 | 22.290 | 32.870 | 36.950 | 42.195 | 54.669 | 55.848 |
|  | Relative Retention Time | 0.33 | 0.37 | 0.48 | 0.56 | 0.61 | 0.68 | 1.00 | 1.12 | 1.28 | 1.66 | 1.70 |
| 210 | Retention Time | 10.932 | 12.324 | 16.003 | 18.568 | 20.082 | 22.399 | 32.993 | 37.063 | 42.330 | 54.839 | 56.012 |
|  | Relative Retention Time | 0.33 | 0.37 | 0.49 | 0.56 | 0.61 | 0.68 | 1.00 | 1.12 | 1.28 | 1.66 | 1.70 |
| 212 | Retention Time | 10.863 | 12.278 | 15.894 | 18.440 | 19.983 | 22.272 | 32.845 | 36.940 | 42.162 | 54.626 | 55.806 |
|  | Relative Retention Time | 0.33 | 0.37 | 0.48 | 0.56 | 0.61 | 0.68 | 1.00 | 1.12 | 1.28 | 1.66 | 1.70 |
| Relative Retention Time RSD/% | | 0 | 0 | 1.2 | 0 | 0 | 0 | 0 | 0 | 0 | 0 | 0 |

**Table M24. Robustness test data (different flow rates)**

| Flow Rate (mL/min)  Time | | Peak 1 | Peak 2 | Peak 3 | Peak 4 | Peak 5 | Peak 6 | Peak 7(S) | Peak 8 | Peak 9 | Peak 10 | Peak 11 |
| --- | --- | --- | --- | --- | --- | --- | --- | --- | --- | --- | --- | --- |
| 0.7 | Retention Time | 12.101 | 13.780 | 17.479 | 20.044 | 21.720 | 24.024 | 34.205 | 38.551 | 43.779 | 56.353 | 57.848 |
|  | Relative Retention Time | 0.35 | 0.40 | 0.51 | 0.59 | 0.63 | 0.70 | 1.00 | 1.13 | 1.28 | 1.65 | 1.69 |
| 0.8 | Retention Time | 10.932 | 12.324 | 16.003 | 18.568 | 20.082 | 22.399 | 32.993 | 37.063 | 42.330 | 54.839 | 56.012 |
|  | Relative Retention Time | 0.33 | 0.37 | 0.49 | 0.56 | 0.61 | 0.68 | 1.00 | 1.12 | 1.28 | 1.66 | 1.70 |
| 0.9 | Retention Time | 9.812 | 11.056 | 14.512 | 17.004 | 18.466 | 20.743 | 31.663 | 35.577 | 40.859 | 53.167 | 54.101 |
|  | Relative Retention Time | 0.31 | 0.35 | 0.46 | 0.54 | 0.58 | 0.66 | 1.00 | 1.12 | 1.29 | 1.68 | 1.71 |
| Relative Retention Time RSD/% | | 6.1 | 6.8 | 5.2 | 4.5 | 4.2 | 3.0 | 0 | 0.52 | 0.45 | 0.92 | 0.59 |

**Table M25. Robustness test data (different column temperatures)**

| Column Temperature (℃) 时间  Time | | Peak 1 | Peak 2 | Peak 3 | Peak 4 | Peak 5 | Peak 6 | Peak 7(S) | Peak 8 | Peak 9 | Peak 10 | Peak 11 |
| --- | --- | --- | --- | --- | --- | --- | --- | --- | --- | --- | --- | --- |
| 28 | Retention Time | 10.993 | 12.638 | 16.110 | 18.600 | 20.442 | 22.527 | 32.948 | 37.230 | 42.307 | 54.748 | 55.954 |
|  | Relative Retention Time | 0.33 | 0.38 | 0.49 | 0.56 | 0.62 | 0.68 | 1.00 | 1.13 | 1.28 | 1.66 | 1.70 |
| 30 | Retention Time | 10.932 | 12.324 | 16.003 | 18.568 | 20.082 | 22.399 | 32.993 | 37.063 | 42.330 | 54.839 | 56.012 |
|  | Relative Retention Time | 0.33 | 0.37 | 0.49 | 0.56 | 0.61 | 0.68 | 1.00 | 1.12 | 1.28 | 1.66 | 1.70 |
| 32 | Retention Time | 10.640 | 11.918 | 15.562 | 18.162 | 19.456 | 21.930 | 32.744 | 36.690 | 42.107 | 54.485 | 55.667 |
|  | Relative Retention Time | 0.32 | 0.36 | 0.48 | 0.55 | 0.59 | 0.67 | 1.00 | 1.12 | 1.29 | 1.66 | 1.70 |
| Relative Retention Time RSD/% | | 1.8 | 2.8 | 1.2 | 1.1 | 2.6 | 0.86 | 0 | 0.52 | 0.45 | 0 | 0 |

| column  Time | | Peak 1 | Peak 2 | Peak 3 | Peak 4 | Peak 5 | Peak 6 | Peak 7(S) | Peak 8 | Peak 9 | Peak 10 | Peak 11 |
| --- | --- | --- | --- | --- | --- | --- | --- | --- | --- | --- | --- | --- |
| Agilent | Retention Time | 10.932 | 12.324 | 16.003 | 18.568 | 20.082 | 22.399 | 32.993 | 37.063 | 42.330 | 54.839 | 56.012 |
|  | Relative Retention Time | 0.33 | 0.37 | 0.49 | 0.56 | 0.61 | 0.68 | 1.00 | 1.12 | 1.28 | 1.66 | 1.70 |
| Waters | Retention Time | 12.682 | 13.926 | 17.773 | 20.316 | 22.368 | 24.267 | 33.907 | 39.772 | 43.479 | 56.481 | 38.048 |
|  | Relative Retention Time | 0.37 | 0.41 | 0.52 | 0.60 | 0.66 | 0.72 | 1.00 | 1.17 | 1.28 | 1.67 | 1.12 |
| Shimadzu | Retention Time | 13.048 | 14.835 | 18.172 | 20.697 | 23.525 | 24.879 | 33.990 | 40.775 | 43.921 | 59.146 | 59.785 |
|  | Relative Retention Time | 0.38 | 0.44 | 0.53 | 0.61 | 0.69 | 0.73 | 1.00 | 1.20 | 1.29 | 1.74 | 1.76 |
| Relative Retention Time RSD/% | | 7.4 | 8.7 | 4.1 | 4.5 | 6.2 | 3.8 | 0 | 3.5 | 0.45 | 2.6 | 23.2 |

**Table M26. Robustness test data (different columns)**

2. **Standard Setting**

36 batches of samples were analyzed according to the prescribed sample preparation procedure and chromatographic conditions detailed below, and the corresponding results were subsequently evaluated.

**2.1 Inspection Method:**

The analysis was performed via high-performance liquid chromatography (HPLC) according to the Chinese Pharmacopoeia General Chapter 0512.

The analysis was conducted according to the sample preparation procedure and chromatographic conditions detailed in Section 1.2.6.

**2.2 Test Results**

The relative retention times of the characteristic peaks compared to the reference peak (S) are presented in the table below.

**Table M27. Summary of test results for 36 batches of samples**

| Number of Samples/Relative Retention Time | Peak 1 | Peak 2 | Peak 3 | Peak 4 | Peak 5 | Peak 6 | Peak 7（S） | Peak 8 | Peak 9 | Peak 10 | Peak 11 |
| --- | --- | --- | --- | --- | --- | --- | --- | --- | --- | --- | --- |
| Reference Herbs | 0.327 | 0.371 | 0.478 | 0.556 | 0.603 | 0.674 | 1.000 | 1.127 | 1.285 | 1.663 | 1.700 |
| AZ2024100076 | 0.329 | 0.372 | 0.482 | 0.560 | 0.606 | 0.677 | 1.000 | 1.126 | 1.283 | 1.662 | 1.698 |
| AZ2024100077 | 0.329 | 0.372 | 0.481 | 0.559 | 0.607 | 0.677 | 1.000 | 1.126 | 1.284 | 1.664 | 1.700 |
| AZ2024100078 | 0.329 | 0.373 | 0.481 | 0.558 | 0.605 | 0.676 | 1.000 | 1.125 | 1.284 | 1.661 | 1.698 |
| AZ2024100079 | 0.329 | 0.372 | 0.481 | 0.559 | 0.606 | 0.677 | 1.000 | 1.126 | 1.284 | 1.664 | 1.700 |
| AZ2024100080 | 0.329 | 0.372 | 0.482 | 0.560 | 0.607 | 0.677 | 1.000 | 1.124 | 1.283 | 1.662 | 1.698 |
| AZ2024100081 | 0.330 | 0.372 | 0.482 | 0.560 | 0.607 | 0.677 | 1.000 | 1.127 | 1.284 | 1.662 | 1.698 |
| AZ2024100082 | 0.327 | 0.372 | 0.480 | 0.557 | 0.604 | 0.675 | 1.000 | 1.126 | 1.285 | 1.661 | 1.698 |
| AZ2024100083 | 0.330 | 0.373 | 0.482 | 0.560 | 0.607 | 0.678 | 1.000 | 1.124 | 1.283 | 1.662 | 1.698 |
| AZ2024100084 | 0.330 | 0.373 | 0.482 | 0.560 | 0.607 | 0.677 | 1.000 | 1.125 | 1.285 | 1.664 | 1.700 |
| AZ2024100085 | 0.329 | 0.372 | 0.481 | 0.559 | 0.607 | 0.676 | 1.000 | 1.127 | 1.284 | 1.662 | 1.699 |
| AZ2024100091 | 0.329 | 0.373 | 0.482 | 0.559 | 0.607 | 0.676 | 1.000 | 1.125 | 1.284 | 1.662 | 1.698 |
| AZ2024100092 | 0.330 | 0.373 | 0.481 | 0.559 | 0.607 | 0.676 | 1.000 | 1.125 | 1.284 | 1.663 | 1.699 |
| AZ2024100093 | 0.329 | 0.372 | 0.481 | 0.559 | 0.606 | 0.677 | 1.000 | 1.125 | 1.284 | 1.664 | 1.700 |
| AZ2024100094 | 0.327 | 0.372 | 0.479 | 0.557 | 0.604 | 0.675 | 1.000 | 1.125 | 1.285 | 1.662 | 1.699 |
| AZ2024100095 | 0.330 | 0.373 | 0.483 | 0.561 | 0.608 | 0.677 | 1.000 | 1.124 | 1.283 | 1.661 | 1.698 |
| AZ2024100096 | 0.329 | 0.372 | 0.481 | 0.559 | 0.606 | 0.676 | 1.000 | 1.105 | 1.283 | 1.661 | 1.697 |
| AZ2024100097 | 0.328 | 0.372 | 0.480 | 0.558 | 0.605 | 0.676 | 1.000 | 1.127 | 1.285 | 1.662 | 1.699 |
| AZ2024100098 | 0.327 | 0.371 | 0.480 | 0.558 | 0.604 | 0.675 | 1.000 | 1.127 | 1.285 | 1.661 | 1.699 |
| AZ2024100099 | 0.328 | 0.372 | 0.480 | 0.557 | 0.604 | 0.675 | 1.000 | 1.127 | 1.284 | 1.663 | 1.700 |
| AZ2024100100 | 0.329 | 0.373 | 0.482 | 0.560 | 0.606 | 0.677 | 1.000 | 1.127 | 1.284 | 1.661 | 1.698 |
| AZ2024100101 | 0.329 | 0.372 | 0.481 | 0.559 | 0.606 | 0.676 | 1.000 | 1.124 | 1.284 | 1.661 | 1.697 |
| AZ2024100102 | 0.328 | 0.372 | 0.480 | 0.557 | 0.604 | 0.675 | 1.000 | 1.127 | 1.284 | 1.661 | 1.699 |
| AZ2024100103 | 0.328 | 0.371 | 0.480 | 0.559 | 0.605 | 0.676 | 1.000 | 1.125 | 1.284 | 1.663 | 1.699 |
| AZ2024100104 | 0.329 | 0.372 | 0.482 | 0.559 | 0.606 | 0.676 | 1.000 | 1.123 | 1.284 | 1.662 | 1.697 |
| AZ2024100105 | 0.329 | 0.372 | 0.493 | 0.561 | 0.601 | 0.677 | 1.000 | 1.125 | 1.282 | 1.659 | 1.695 |
| AZ2024100106 | 0.330 | 0.372 | 0.482 | 0.560 | 0.606 | 0.678 | 1.000 | 1.124 | 1.283 | 1.660 | 1.696 |
| AZ2024100107 | 0.329 | 0.373 | 0.481 | 0.558 | 0.605 | 0.675 | 1.000 | 1.128 | 1.285 | 1.662 | 1.700 |
| AZ2024100108 | 0.329 | 0.372 | 0.482 | 0.560 | 0.607 | 0.677 | 1.000 | 1.124 | 1.283 | 1.661 | 1.697 |
| AZ2024100109 | 0.329 | 0.372 | 0.482 | 0.560 | 0.606 | 0.677 | 1.000 | 1.125 | 1.283 | 1.661 | 1.697 |
| AZ2024100110 | 0.328 | 0.373 | 0.480 | 0.557 | 0.604 | 0.674 | 1.000 | 1.127 | 1.284 | 1.661 | 1.698 |
| AZ2024100111 | 0.329 | 0.372 | 0.481 | 0.560 | 0.607 | 0.677 | 1.000 | 1.125 | 1.283 | 1.662 | 1.698 |
| AZ2024100112 | 0.327 | 0.372 | 0.479 | 0.556 | 0.604 | 0.674 | 1.000 | 1.125 | 1.285 | 1.662 | 1.699 |
| AZ2024100114 | 0.327 | 0.372 | 0.480 | 0.558 | 0.605 | 0.675 | 1.000 | 1.125 | 1.286 | 1.663 | 1.700 |
| AZ2024100115 | 0.329 | 0.373 | 0.482 | 0.559 | 0.606 | 0.677 | 1.000 | 1.127 | 1.284 | 1.661 | 1.698 |
| AZ2024100116 | 0.327 | 0.372 | 0.479 | 0.557 | 0.604 | 0.675 | 1.000 | 1.126 | 1.285 | 1.662 | 1.699 |
| AZ2024100117 | 0.327 | 0.372 | 0.479 | 0.557 | 0.604 | 0.675 | 1.000 | 1.126 | 1.285 | 1.663 | 1.700 |
| Mean | 0.33 | 0.37 | 0.48 | 0.56 | 0.61 | 0.68 | 1.00 | 1.12 | 1.28 | 1.66 | 1.70 |

**2.3 Inspection Conclusion**

The characteristic chromatograms of all 36 batches exhibited 11 characteristic peaks, which corresponded to the 11 characteristic peaks observed in the reference crude drug material chromatogram. Among these, the retention times of Peak 2, Peak 3, Peak 4, Peak 6, Peak 7, Peak 8, and Peak 9 were consistent with those of the reference standards for protocatechuic acid, coniferin, syringin, eleutheroside B1, eleutheroside E, isofraxidin, and syringaresinol-4'-O-*β*-D-glucopyranoside, respectively. Using the peak corresponding to the eleutheroside E reference standard as the S peak, the relative retention times of each characteristic peak relative to the S peak should be within ±10% of the specified values. The specified relative retention times are as follows: 0.33 (Peak 1), 0.37 (Peak 2), 0.48 (Peak 3), 0.56 (Peak 4), 0.61 (Peak 5), 0.68 (Peak 6), 1.12 (Peak 8), 1.28 (Peak 9), 1.66 (Peak 10), and 1.70 (Peak 11).

**Supporting figures and tables:**

**Table S1. The contribution values of various environmental factors obtained from the initial maxent run results**

| **Variable** | **Percent contribution** | **Permutation importance** |
| --- | --- | --- |
| bio18 | 36.2 | 6.6 |
| Ndvi | 15.1 | 4.1 |
| bio11 | 12.7 | 1.8 |
| bio01 | 7.7 | 45.4 |
| Human | 7.6 | 3.8 |
| bio04 | 5.4 | 5.6 |
| bio16 | 3.7 | 17.4 |
| silt | 2.2 | 1.6 |
| bio13 | 1.8 | 1.1 |
| bio09 | 1.5 | 5 |
| ph | 1.1 | 1.4 |
| bio08 | 1 | 0.6 |
| bio07 | 1 | 0.1 |
| bio06 | 0.6 | 0.4 |
| bio10 | 0.4 | 0.1 |
| bio02 | 0.4 | 0.5 |
| bio15 | 0.3 | 0.3 |
| clay | 0.3 | 0.8 |
| bio03 | 0.2 | 0.3 |
| bio14 | 0.2 | 1.2 |
| bio05 | 0.2 | 0.7 |
| soc | 0.1 | 0.2 |
| bio19 | 0.1 | 0.3 |
| bio17 | 0.1 | 0.3 |
| bulk | 0.1 | 0.3 |
| bio12 | 0 | 0.1 |

**Table S2 Analysis of the Effect of Different Pseudo-absence Points on Model Accuracy**

| **Models** | **Number of Pseudo-absence Points** | | | |
| --- | --- | --- | --- | --- |
|  | **229** | **1000** | **5000** | **10000** |
| SRE | 0.619933333 | 0.674933333 | **0.678800000** | 0.602888889 |
| RF | 0.940266667 | **0.948066667** | 0.942600000 | 0.944777778 |
| MAXENT | 0.862066667 | 0.777600000 | 0.936733333 | **0.946222222** |
| MARS | 0.925066667 | 0.948333333 | 0.941466667 | **0.950000000** |
| GLM | 0.919133333 | 0.938066667 | 0.935533333 | **0.941666667** |
| GBM | 0.940200000 | 0.947866667 | **0.957333333** | 0.955000000 |
| GAM | 0.888866667 | 0.869333333 | 0.900400000 | **0.901666667** |
| FDA | **0.936666667** | 0.931533333 | 0.900533333 | 0.900666667 |
| CTA | 0.890133333 | 0.904866667 | **0.907066667** | 0.906333333 |
| ANN | 0.893600000 | 0.897200000 | **0.897466667** | 0.870000000 |
| Mean | 0.881593333 | 0.883780000 | **0.899793333** | 0.891922222 |

**Fig. S1. PCA of Multidimensional Climatic and Soil Environmental Variables.** (A) PCA of climate variables related to temperature; (B) PCA of climate variables associated with moisture; (C) PCA of soil variables characterizing physical properties; (D) PCA of soil variables reflecting chemical properties. Color gradients indicate the relative importance of each variable.

**Fig. S2. Layers of different principal components derived from the Principal Component Analysis (PCA) of temperature-related climatic variables under current conditions.**

**Fig. S3. Layers of different principal components derived from the Principal Component Analysis (PCA) of humidity-related climatic variables under current conditions.**

**Fig. S4. Layers of different principal components derived from the Principal Component Analysis (PCA) of soil environmental variables related to physical properties under current climatic conditions**

**Fig. S5. Layers of different principal components derived from the Principal Component Analysis (PCA) of soil environmental variables related to chemical properties under current climatic conditions**

**Table S3 Projected changes in the dominant factors within the current suitable habitat of E. senticosus in Heilongjiang Province under future climate scenarios**

| Factors | GLM | GBM | GAM | CTA | ANN | SRE | FDA | MARS | RF | MAXENT | Mean |
| --- | --- | --- | --- | --- | --- | --- | --- | --- | --- | --- | --- |
| Bio18 | 0.474 | 0.647 | 0.732 | 0.776 | 0.893 | 0.677 | 0.207 | 0.566 | 0.133 | 0.420 | 0.553 |
| Bio11 | 0.551 | 0.381 | 0.535 | 0.575 | 0.525 | 0.463 | 0.102 | 0.361 | 0.113 | 0.407 | 0.401 |
| NDVI | 0.395 | 0.033 | 0.218 | 0.066 | 0.101 | 0.473 | 0.377 | 0.330 | 0.101 | 0.378 | 0.247 |
| Bio08 | 0.069 | 0.133 | 0.511 | 0.251 | 0.110 | 0.471 | 0.019 | 0.103 | 0.054 | 0.076 | 0.180 |
| Bio15 | 0.122 | 0.012 | 0.297 | 0.035 | 0.229 | 0.109 | 0.008 | 0.021 | 0.013 | 0.070 | 0.092 |
| Silt | 0.031 | 0.003 | 0.283 | 0.000 | 0.144 | 0.207 | 0.041 | 0.078 | 0.062 | 0.057 | 0.091 |
| Bio19 | 0.079 | 0.001 | 0.234 | 0.000 | 0.309 | 0.124 | 0.000 | 0.027 | 0.009 | 0.009 | 0.079 |
| Ph | 0.026 | 0.004 | 0.379 | 0.033 | 0.041 | 0.221 | 0.000 | 0.000 | 0.003 | 0.014 | 0.072 |
| Human | 0.060 | 0.051 | 0.045 | 0.064 | 0.074 | 0.213 | 0.023 | 0.053 | 0.016 | 0.103 | 0.070 |
| Bio14 | 0.049 | 0.001 | 0.228 | 0.010 | 0.117 | 0.156 | 0.004 | 0.008 | 0.010 | 0.018 | 0.060 |
| Clay | 0.036 | 0.000 | 0.236 | 0.000 | 0.076 | 0.126 | 0.022 | 0.062 | 0.003 | 0.026 | 0.059 |
| Bio02 | 0.027 | 0.004 | 0.086 | 0.002 | 0.064 | 0.193 | 0.003 | 0.003 | 0.010 | 0.065 | 0.046 |
| Soc | 0.008 | 0.000 | 0.157 | 0.000 | 0.025 | 0.211 | 0.000 | 0.000 | 0.004 | 0.003 | 0.041 |

**Table S4 Projected changes in the dominant factors within the current suitable habitat of *E. senticosus* in Heilongjiang Province under future climate scenarios**

| Environmental  factor | Climate scenario combinations | | 95% | | Mean |
| --- | --- | --- | --- | --- | --- |
|  | Years | Carbon emission | 2.5% | 97.5% |  |
| Bio18 | Current | | 303.437 | 405.287 | 347.716 |
|  | 2050s | SSP126 | 331.333 | 448.341 | 385.562 |
|  |  | SSP245 | 327.659 | 437.667 | 377.709 |
|  |  | SSP370 | 316.333 | 429.008 | 369.623 |
|  |  | SSP585 | 328.000 | 444.000 | 380.318 |
|  | 2070s | SSP126 | 337.667 | 447.000 | 386.844 |
|  |  | SSP245 | 337.000 | 457.333 | 391.681 |
|  |  | SSP370 | 323.659 | 438.341 | 377.977 |
|  |  | SSP585 | 337.000 | 457.333 | 391.552 |
|  | 2090s | SSP126 | 337.325 | 453.333 | 391.557 |
|  |  | SSP245 | 337.333 | 460.667 | 394.539 |
|  |  | SSP370 | 329.000 | 447.000 | 384.704 |
|  |  | SSP585 | 341.992 | 469.667 | 401.359 |
| Bio11 | Current | | -22.390 | -14.138 | -17.849 |
|  | 2050s | SSP126 | -19.633 | -11.367 | -15.096 |
|  |  | SSP245 | -19.200 | -11.132 | -14.779 |
|  |  | SSP370 | -19.133 | -11.232 | -14.816 |
|  |  | SSP585 | -18.500 | -10.567 | -14.164 |
|  | 2070s | SSP126 | -19.733 | -11.467 | -15.201 |
|  |  | SSP245 | -18.633 | -10.600 | -14.212 |
|  |  | SSP370 | -17.833 | -10.000 | -13.548 |
|  |  | SSP585 | -16.833 | -8.933 | -12.493 |
|  | 2090s | SSP126 | -19.600 | -11.367 | -15.076 |
|  |  | SSP245 | -18.200 | -10.167 | -13.789 |
|  |  | SSP370 | -16.267 | -8.567 | -12.036 |
|  |  | SSP585 | -14.533 | -6.732 | -10.237 |
| Bio08 | Current | | 16.503 | 21.618 | 19.158 |
|  | 2050s | SSP126 | 20.933 | 25.167 | 23.361 |
|  |  | SSP245 | 21.033 | 25.267 | 23.483 |
|  |  | SSP370 | 20.967 | 25.167 | 23.382 |
|  |  | SSP585 | 21.633 | 25.867 | 24.066 |
|  | 2070s | SSP126 | 21.000 | 25.233 | 23.451 |
|  |  | SSP245 | 21.567 | 25.800 | 24.013 |
|  |  | SSP370 | 21.833 | 26.033 | 24.245 |
|  |  | SSP585 | 22.967 | 27.233 | 25.440 |
|  | 2090s | SSP126 | 20.900 | 25.100 | 23.327 |
|  |  | SSP245 | 21.933 | 26.167 | 24.348 |
|  |  | SSP370 | 22.933 | 27.133 | 25.351 |
|  |  | SSP585 | 24.367 | 28.567 | 26.822 |

**Fig. S6. Spatial distribution patterns of the comprehensive chemical composition index of *E. senticosus* in Heilongjiang Province under various combined climate scenarios.**

**Fig. S7. Spatial distribution patterns of coniferin content in *E. senticosus* within Heilongjiang Province under various combined climate scenarios.**

**Fig. S8. Spatial distribution patterns of syringin content in *E. senticosus* within Heilongjiang Province under various combined climate scenarios.**

**Fig. S9. Spatial distribution patterns of isofraxidin content in *E. senticosus* within Heilongjiang Province under various combined climate scenarios.**

**Fig. S10. Spatial distribution patterns of syringaresinol-4'-O-*β*-D-glucopyranoside content in *E. senticosus* within Heilongjiang Province under various combined climate scenarios.**

**Table S5 Nature Reserve Number**

| Number | Name |
| --- | --- |
| 1 | Mudanjiang Jingpo Lake Scenic Area |
| 2 | Shengshan National Nature Reserve |
| 3 | Phoenix Mountain National Forest Park |
| 4 | Jinlongshan National Forest Park |
| 5 | Xiaoxing'an Mountains National Park, Yichun City, Heilongjiang Province |
| 6 | Heilongjiang Xiaobeihu Nature Reserve |
| 7 | Bali Bay National Forest Park |
| 8 | Wulong National Forest Park |
| 9 | Muling Northeast Taxus chinensis National Nature Reserve |
| 10 | Jilin Huangnihe National Nature Reserve |
| 11 | Boli National Forest Park |
| 12 | Jiejinshan National Forest Park |
| 13 | Sandaoguan National Forest Park |
| 14 | Yabuli National Forest Park |
| 15 | Changshoushan National Forest Park |
| 16 | Yimianpo National Forest Park |
| 17 | Heilongjiang Shankou Geological Park |
| 18 | Mudanfeng National Forest Park |
| 19 | Wuyiling National Nature Reserve |
| 20 | Raohe Northeast Black Bee National Nature Reserve |
| 21 | Xinqing White-headed Crane Nature Reserve |
| 22 | Qingshan National Forest Park |
| 23 | Heilongjiang Jiagedaqi National Forest Park |
| 24 | Heilongjiang Nanbei River Wetland Nature Reserve |
| 25 | Changshou National Forest Park |
| 26 | Danqinghe National Forest Park |
| 27 | Jinquan National Forest Park |
| 28 | Huzhong National Forest Park |
| 29 | Plum Hill National Forest Park |

**Fig. S11. Priority counties suitable for the wild tending of *E. senticosus* targeting high comprehensive chemical composition under future climate scenarios.**

**Fig. S12. Priority counties suitable for the wild tending of *E. senticosus* targeting high coniferin content under future climate scenarios**

**Fig. S13. Priority counties suitable for the wild tending of *E. senticosus* targeting high syringin content under future climate scenarios**

**Fig. S14. Priority counties suitable for the wild tending of *E. senticosus* targeting high isofraxidin content under future climate scenarios.**

**Fig. S15. Priority counties suitable for the wild tending of *E. senticosus* targeting high syringaresinol-4'-O-*β*-D-glucopyranoside content under future climate scenarios.**
